# Reactive Oxygen Species Partly Mediate DNA Methylation in Responses to Different Heavy Metals in Pokeweed

**DOI:** 10.3389/fpls.2022.845108

**Published:** 2022-04-07

**Authors:** Minyu Jing, Hanchao Zhang, Mingyue Wei, Yongwei Tang, Yan Xia, Yahua Chen, Zhenguo Shen, Chen Chen

**Affiliations:** ^1^College of Life Sciences, Nanjing Agricultural University, Nanjing, China; ^2^Jiangsu Collaborative Innovation Center for Solid Organic Waste Resource Utilization, Nanjing Agricultural University, Nanjing, China

**Keywords:** heavy metal, pokeweed, reactive oxygen species, DNA methylation, methylase and demethylase

## Abstract

DNA methylation is a rapid response strategy promoting plant survival under heavy metal (HM) stress. However, the roles of DNA methylation underlying plant adaptation to HM stress remain largely unknown. Here, we used pokeweed, a hyperaccumulator of manganese (Mn) and cadmium (Cd), to explore responses of plant to HM stress at phenotypic, transcriptional and DNA methylation levels. Mn- and Cd-specific response patterns were detected in pokeweed. The growth of pokeweed was both inhibited with exposure to excess Mn/Cd, but pokeweed distinguished Mn and Cd with different subcellular distributions, ROS scavenging systems, transcriptional patterns including genes involved in DNA methylation, and differentially methylated loci (DML). The number of DML between Mn/Cd treated and untreated samples increased with increased Mn/Cd concentrations. Meanwhile, pretreatment with NADPH oxidase inhibitors prior to HM exposure markedly reduced HM-induced reactive oxygen species (ROS), which caused reductions in expressions of DNA methylase and demethylase in pretreated samples. The increased levels of HM-induced demethylation were suppressed with alleviated ROS stress, and a series of HM-related methylated loci were also ROS-related. Taken together, our study demonstrates that different HMs affect different DNA methylation sites in a dose-dependent manner and changes in DNA methylation under Mn/Cd stress are partly mediated by HM-induced ROS.

## Introduction

Heavy metal contamination in soils is a critical environmental issue, which has direct and detrimental impacts on plant growth (Chibuike and Obiora, 2014; Tóth et al., 2016). To promote survival in metalliferous soils, some plants have evolved a series of protective mechanisms that enhance heavy metal tolerance (Singh et al., 2016). Among them, hyperaccumulator plants not only grow well on metalliferous soils but also allow the entrance of extraordinarily high concentrations of heavy metals in their aerial organs (Fasani et al., 2017). Such hyperaccumulators have been important models to explore the mechanisms underlying adaptation of plants to heavy metal environments.

Previous studies on heavy metal tolerance of hyperaccumulators mainly focused on genetic elements. Various genes have been reported to involve in internal detoxification and sequestration (Liu et al., 2017; Chaudhary et al., 2018; Zhang et al., 2020). Recent studies have found that some hyperaccumulators with limited genetic variation among populations still have a wide range of phenotypes in different metalliferous habitats (Chen et al., 2015). Epigenetic variation which can regulate phenotypic variation could be one potentially important way for plant in response to environmental changes (Niederhuth and Schmitz, 2014; Wibowo et al., 2016; Li et al., 2021). DNA methylation is one of the main epigenetic mechanisms enabling adaptation of plants in complex environments (Niederhuth and Schmitz, 2014; Chang et al., 2019; Perrone and Martinelli, 2020; Li et al., 2021; Chen et al., 2022). Changes of DNA methylation in response to environmental stresses can be relatively stable, and establish a DNA methylation-dependent stress memory to the offspring (Wibowo et al., 2016; Williams and Gehring, 2017; Sudan et al., 2018). A growing body of evidences has demonstrated that DNA methylation may be a rapid response strategy promoting plant survival under heavy metal stress (Ou et al., 2012; Ding et al., 2014; Shi et al., 2017; Greco et al., 2019).

In plants, genomic DNA is methylated at cytosine in three distinct sequence contexts: cytosine-guanine (CG), cytosine-H-guanine (CHG), and cytosine-H-H (CHH), where H indicates adenine (A), cytosine (C), or thymine (T). DNA methylation or demethylation is catalyzed by a subset of enzymes (Zhang et al., 2018). Cytosine modifications in the CG context are maintained by methyltransferase 1 (MET1), modifications in the CHG context are maintained by chromomethylase 2 (CMT2) or 3 (CMT3), and modifications in the CHH context are maintained by domains rearranged methylase 2 (DRM2) or CMT2 (Bartels et al., 2018). DNA demethylation is catalyzed by a range of DNA glycosylases, including repressor of silencing 1 (ROS1), demeter (DME), and demeter-like 2 and 3 (DML2 and DML3; Li et al., 2018). It has been reported that DNA methyltransferases and glycosylases are differentially expressed under heavy metal stress which further lead to differentially methylated loci (Gullì et al., 2018; Ferrari et al., 2020). DNA methylation in gene body usually enhances genetic transcription, and DNA methylation in gene promoters and transposable elements is correlated to gene and transposon silencing (Zhang et al., 2006, 2018; Feng et al., 2016; Bewick and Schmitz, 2017). Besides, the environmental stress can cause changes in gene expression through hypomethylation or hypermethylation of DNA (Chinnusamy and Zhu, 2009; Chen et al., 2022). Furthermore, the methylation variation induced by heavy metal stress might be functional as transgenerational memory (Ou et al., 2012; Cong et al., 2019). Thus, DNA methylation is particularly important in defining signal specificity associated with heavy metal tolerance and contributes to the adaptation to heavy metal stress (Ferrari et al., 2020). Indeed, changes of gene expressions under heavy metal stress have been reported to involve in variation in DNA methylation level (Singh et al., 2021). Until now, the prevailing view is that the heavy metal stress increases DNA methylation of plant at the genome level (Ding et al., 2014; Feng et al., 2016; Fan et al., 2020). However, recent studies have reported that some heavy metal stress might lead to increased demethylation (Peng and Zhang, 2009; Dowen et al., 2012; Yaish, 2013), suggesting that DNA methylation levels may differ under excess levels of different metals, even within same species. For example, Cong et al. (2019) observed that hypomethylation was induced under excess amounts of cadmium (Cd), chromium (Cr), and copper (Cu), whereas no changes of DNA methylation were detected in rice exposing to mercury (Hg). Therefore, both hypomethylation and hypermethylation may occur in response to excess levels of metals with different properties. However, the detailed responses of plants to stress caused by different heavy metals at DNA methylation level are still largely unknown.

Reactive oxygen species (ROS) are general toxicity mechanisms implicated in plant responses to heavy metal stress, which can be triggered directly or indirectly by heavy metals (Chibuike and Obiora, 2014; Shahid et al., 2014). Excessive ROS productions could lead to imbalances between the ROS production and their scavenging systems, triggering oxidative stress and resulting in deleterious effects such as lipid peroxidation and DNA lesions (Choudhury et al., 2017; Sharma et al., 2022). A few studies have reported that ROS may result in increased DNA demethylation (Aina et al., 2004; Yaish, 2013). However, the effects of metal-induced ROS on DNA methylation patterns remain poorly understood. Besides, although we know that heavy metal stress could disturb DNA methylation, the relationship among heavy metals, oxidative stress and DNA methylation is unclear.

Pokeweed (*Phytolacca americana* L.) is a perennial herb that thrives in heavy metal-contaminated habitats (Chen et al., 2015). Pokeweed has been reported as both manganese (Mn) and Cd hyperaccumulator, and can be used for phytoremediation (Peng et al., 2008; Gao et al., 2013; Chen et al., 2015, 2022). Mn is an essential element which is necessary for plant growth as a component of many enzymes, while Cd is non-essential element. High concentrations of both Mn and Cd can lead to inhibition of plant growth and development (Rascio and Navari-Izzo, 2011). The ability of pokeweed to flourish in Mn and Cd contaminated habitats is due to its high tolerance to both metals, despite their differing physiological functions and effects. Thus, pokeweed could be a good model to explore the DNA methylation patterns under stress caused by different types of heavy metals (Chen et al., 2022). Meanwhile, the role of metal-induced ROS in DNA methylation variation can be investigated.

In this study, we adopted methylation-sensitive amplification polymorphism profiling (MSAP) and RNA sequencing (RNA-seq) to identify changes of DNA methylation pattern in pokeweed seedlings exposing to a range of concentrations of Mn, Cd, and NADPH oxidase inhibitors. Isogenic pokeweeds were used to eliminate effects associated with genetic variation. Combined with phenotypes, epigenome-wide association analysis (EWAS) was performed to examine the correlations between methylated loci and contents of Mn/Cd or ROS. Our study focused on relationships among epigenetic, transcriptional and phenotypic variation of hyperaccumulators in different heavy metal habitats, which will improve our understanding of the rapid adaptation mechanism(s) of plants under heavy metal stress.

## Materials and Methods

### Plant Materials and Chemical Treatments

Pokeweed seeds were harvested from a lead and zinc ore smeltery in Jishou, Hunan Province, China (JS, 28°17′49″N, 109°45′7″E; Chen et al., 2015). Healthy, plump, and uniform pokeweed seeds were selected, soaked in concentrated sulfuric acid (mass fraction >70%) for 10 min and then rinsed thoroughly with sterilized water. Seeds were soaked in tap water for 15 days and germinated in vermiculite on plastic plates. Following germination, seedlings were transplanted to plastic pots containing 2.5 L Hoagland nutrient solution and grown for 10 days (Hoagland and Arnon, 1950). The pH of the nutrient solution was adjusted to 5.5, and the nutrient solution was replaced every 2 days. Seedlings were grown in standard greenhouse conditions at 25°C under a 13/11 h day/night photoperiod. Uniform seedlings were subjected to one of 10 treatments for 20 days, including five Mn treatments (9 μM, 2 mM, 5 mM, 10 mM, and 20 mM), four Cd treatments (5, 10, 50, and 100 μM), and one untreated control. To assess oxidative stress resulting from the Mn and Cd treatments, a separate batch of seedlings was pretreated for 16 h with either diphenyleneiodonium chloride (DPI) or imidazole (IMZ), both of which are NADPH oxidase inhibitors, prior to 2 days of heavy metal exposure. Treatment combinations included: (i) 20 mM Mn, (ii) 20 mM Mn + 10 μM DPI, (iii) 20 mM Mn + 500 μM IMZ, (iv) 50 μM Cd, (v) 50 μM Cd + 10 μM DPI, (vi) 50 μM + 500 μM IMZ, and (vii) sterile water (control). At the end of the treatment period, we harvested plants and measured shoot height, length of the longest main root, and fresh weight. Roots were soaked in 10 mM CaCl_2_ solution for 20 min and rinsed thoroughly with deionized water to remove metal ions from the root surface. A portion of the freshly harvested material was used in physiological analysis, and the other portion was stored in a freezer at −80°C for further analyses. Three or six biological replicates from each treatment were used in morphological analysis.

### Phenotypic Analysis

We determined the distribution of accumulated Cd and Mn in leaf cells. Photosynthetic pigment content including chlorophyll *a*, chlorophyll *b*, and total carotenoid content was determined, and photosynthetic gas exchange parameters were measured. The content of hydrogen peroxide (H_2_O_2_), a common ROS, was measured. Lipid peroxidation was measured by assessing the reaction of malondialdehyde (MDA) to thiobarbituric acid (TBA). The content of 8-hydroxy-2′-deoxyguanosine (8-OHdG) used as a proxy for DNA damage was assessed. Enzymatic antioxidant activity of pokeweed was investigated by carrying out superoxide dismutase (SOD), peroxidase (POD) and catalase (CAT) activity assays. The detailed methods for phenotypic analysis are provided in Supplementary Material.

### Transcriptome Sequencing and Gene Expression Analysis

Assessment of differential gene expression at the transcription level was performed using three treatments, including sterile water (control), 20 mM Mn, and 50 μM Cd. Each treatment included three biological replicates, which were used in both expression profile analysis and quantitative real time PCR (qRT-PCR) analysis. Total RNA was extracted from the antepenultimate leaf of each replicate using a plant RNA extraction kit (Takara, Dalian, China), and genomic DNA was extracted using DNase I (Takara). RNA quality was analyzed using an Agilent 2100 Bioanalyzer (Agilent Technologies) and subsequently quantified using a Nanodrop 2000 spectrophotometer (Thermo Fisher Scientific, Waltham, MA, United States). Nine RNA samples acquired from the three treatments were used to construct RNA-seq libraries for expression profile analysis. In addition, equal amounts of RNA from each treatment were mixed for transcriptome sequencing, and this assembly transcriptome was used as a reference in expression profile analysis. RNA-seq libraries were constructed following standard procedures using a TruSeq RNA Library Prep Kit (Illumina, San Diego, CA, United States). Libraries were sequenced with a HiSeq 2500 System (Illumina) at Biozeron Biotechnology in Shanghai. The raw sequencing reads are available under NCBI BioProject accession no. PRJNA623405. Adapters and low-quality reads were removed using Trimmomatic (Bolger et al., 2014), and remaining reads were assembled *de novo* using default parameters in Trinity (Haas et al., 2013). UniGene annotation was performed using a BLASTx search against the NR, Swiss-Prot, KEGG, and COG protein databases. Clean reads were mapped to the reference transcriptome sequences for expression profile analysis, and gene expression was obtained using RSEM (Li and Dewey, 2011). Identification of differentially expressed genes (DEGs) was then performed on FPKM values using Cuffdiff (Ghosh and Chan, 2016) with a false discovery rate of <0.05 and |log2FC| ≥1. Twenty-one DEGs responding to Mn or Cd stress were randomly selected to verify the reliability of transcriptome analysis by qRT-PCR (Supplementary Tables S1, S2).

To explore the gene expression of DNA methyltransferases and glycosylases under heavy metal stress, we performed qRT-PCR analysis on homologs of CMT2, CMT3, MET1, and ROS1. Meanwhile, the expressions of two genes related to NADPH oxidase (NQO1 and RBOHH) were analyzed. Primer pairs were designed using Primer 5.0 (PRIMER-e, Auckland, NZ; Supplementary Table S2). RNA was reverse transcribed using a PrimeScript RT Reagent Kit with gDNA Eraser (Takara). We conducted qRT-PCR using a Mastercycler ep realplex2 (Eppendorf, Hamburg, Germany). Alpha-3 and -5 tubulin were used as endogenous controls. All qRT-PCR runs were performed three times using three biological replicates. We assessed PCR specificity using melting curve analysis, and analyzed data using the 2^–ΔΔCt^ method (Schmittgen and Livak, 2008).

### Methylation-Sensitive Amplification Polymorphism Profiling

We used the methylation-sensitive AFLP (MSAP) method of Reyna-López et al. (1997) with slight modification (Chen et al., 2020) to analyze epigenetic variation. A total of 102 samples from all treatments were used in MSAP profiling, including samples pretreated with NADPH oxidase inhibitors. Each treatment included six biological replicates. Total genomic DNA was extracted using DNA Plantzol Reagent (Invitrogen, Carlsbad, CA, United States) according to manufacturer protocols. Genomic DNA was double digested with *EcoR*I/*Hpa*II or *EcoR*I/*Msp*I, followed by pre-amplification and selective amplification. *Hpa*II and *Msp*I are methylation sensitive, as identified by the tetranucleotide sequence 5′-CCGG-3′s, but they have different sensitivities to cytosine methylation. Cleavage by *Hpa*II is blocked when the outer or inner cytosine is methylated on both strands, whereas cleavage by *Msp*I is blocked when the outer cytosines are hemi-methylated or fully methylated. Eight fluorescent-labeled primer pairs were used in selective amplification (Supplementary Table S3), and the products were resolved by Genewiz, Inc. (Suzhou, China) using an ABI 3730 DNA Sequencer (Thermo Fisher Scientific). Fragments of 69–450 bp were identified using GENEMARKER ver. 2.2.0 (SoftGenetics, State College, PA, United States), and epigenetic fragments were transformed into a binary character matrix. Four types of methylation were defined: Type 1, a fragment present in *Msp*I and *Hpa*II (1,1), indicated no methylation; Type 2, a fragment present in *Hpa*II but absent in *Msp*I (1,0), indicated hemimethylation of internal or external cytosines; Type 3, a fragment present in *Msp*I but absent in *Hpa*II (0,1), indicated full methylation of internal cytosines; and Type 4, a fragment absent in both *Msp*I and *Hpa*II (0,0), indicated either full methylation of external or both cytosines, or hemimethylation of both cytosines/genetic polymorphism sites (Schulz et al., 2013). Besides, Type 2 + 3 were treated as total methylation. Then, a MSAP profile was constructed to compute epigenetic parameters using “Mixing Scoring 1” method (Schulz et al., 2013; Supplementary Material).

### Statistical Analyses

We quantified epigenetic diversity using the *msap* package in *R* to calculate the frequencies of methylation Types 1–4 and the number of methylation-susceptible loci (MSL; Schulz et al., 2013). Pairwise Nei’s epigenetic distance was calculated using with GENALEX software (ver. 6.5; Peakall and Smouse, 2012).

To investigate the association between methylated loci (nonmethylated loci and total methylated loci) and phenotypes (i.e., Mn, Cd, and H_2_O_2_ contents), we performed an epigenome-wide association analysis (EWAS) using a generalized linear model (GLM) in TASSEL 5.2.52 (Bradbury et al., 2007). According to Price et al. (2006), we used a principal component analysis (PCA) rather than a Q-matrix to summarize genome-wide patterns of relatedness. Because the seeds used in this study were sampled from a single population, they did not conform to Hardy–Weinberg equilibrium; thus, the STRUCTURE algorithm was not suitable for estimating population epigenetic structure and calculating the Q-matrix. We therefore opted for the PCA approach instead of using Q (Zhao et al., 2005). PCA was generated in TASSEL 5.2.52 by using methylated loci. We included epigenotypic, phenotypic, and PCA data in the GLM. Significance of loci-trait associations was defined as *p* < 0.05. These significantly trait associated loci were treated as differentially methylated loci (DML). Manhattan plots were created using the *ggplot2* package in R (Wickham, 2009), and Venn diagrams were created in BioVenn.^1^

Principal coordinate analysis (PCoA) was used to visualize the epigenetic structure of samples treated with Mn/Cd concentration gradient. Bray–Curtis dissimilarity indexes of PCoA were calculated based on the MSAP matrices using the vegdist function in the *vegan* package (Khor et al., 2016) and then the PCoA plots were generated using the *ggplot2* package in R (Wickham, 2009). Redundancy analysis (RDA) was performed using the *rda* function in *vegan* (Oksanen et al., 2019) to evaluate the relative importance of heavy metals or oxidative stress biomarkers as predictors of epigenetic divergence among treatments. We used the formula rda (*x* ~ *y*), where *x* = MSAP profile and *y* = explanatory variables. The explanatory variables comprised the Mn/Cd contents in the three cellular components (F1–F3) or contents of oxidative stress biomarkers, including MDA, H_2_O_2_, and 8-OHdG. We used the *envfit* function with 999 permutations to test the significance of each explanatory variable. Here, R^2^ represents the coefficient of determination, and value of *p* indicate the significance of correlations. Besides, we evaluated the correlation between epigenetic variation and DEG expression change using the following formula: capscale (*x* ~ *y*) where *x* = the Euclidean distance matrix for FPKM values of DEGs as the dependent variable, and *y* = the first eight PCA axes of MSAP profile (MSAP-PC1, MSAP-PC2, MSAP-PC3, MSAP-PC4, MSAP-PC5, MSAP-PC6, MSAP-PC7, MSAP-PC8) as predictors of DEGs variation. The PCs were derived from PCA using the “prcomp” function in *R* with the following formula: prcomp (*r*) where *r* = the binary character matrix for MSAP profile treated with Mixed Scoring 1. The eight PCs totally explained 100% of the variation of MSAP profile. Phenotypic and qRT-PCR data were analyzed using a one-way analysis of variance (ANOVA) in SPSS version 22.0 (IBM, Armonk, NY, United States). Significant differences were determined using Duncan’s multiple range tests at *p* < 0.05.

## Results

### Differential Phenotypic Responses to Mn and Cd by Pokeweed

**Figure 1:**
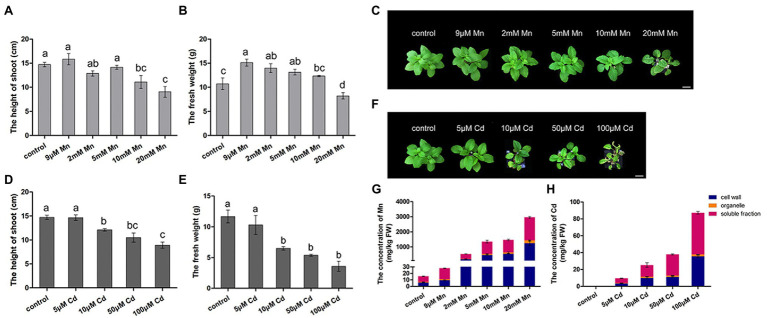
Differential responses to Mn and Cd by pokeweed at phenotypic level. **(A**–**C)** Growth of seedlings subject to Mn treatments: **(A)** shoot height; **(B)** fresh weight; **(C)** photos showing growth; bar = 5 cm. **(D**–**F)** Growth of seedlings subject to Cd treatments: **(D)** shoot height; **(E)** fresh weight; **(F)** photos showing growth; bar = 5 cm. **(G)** Mn concentrations in cell walls (F1), organelles (F2), and the soluble fraction (F3); and **(H)** Cd concentrations in the same three leaf components. Data represent mean ± SD of three biological replicates per treatment for (**A**–**E**) and six biological replicates per treatment for **(G,H**). Letter annotations indicate significant differences according to Duncan’s multiple range test (*p* < 0.05).

We firstly compared the different phenotypic responses of pokeweed to Mn and Cd stress. Both excessive levels of Mn and Cd resulted in reductions in shoot height and fresh weight of pokeweed (Figures 1A–F). Consistently, content of photosynthetic pigments and photosynthetic gas exchange parameters significantly decreased in treatments with excess Mn or Cd (20 mM Mn or 50 μM Cd; Supplementary Figure S1). However, we detected different responses of pokeweed to Mn and Cd which were at low levels. The growth of pokeweed was promoted under low levels of Mn treatments (9 μM), whereas the growth of pokeweed was inhibited under low levels of Cd treatments (10 μM). We further analyzed the accumulation and subcellular distribution of Mn and Cd in pokeweed (Figures 1G,H). Levels of cellular Mn and Cd increased with the increasing of Mn and Cd concentrations, and both two metals accumulated primarily in cell walls and the soluble fraction (Figures 1G,H). But different responses of pokeweed to Mn and Cd at subcellular distribution were detected. Proportion of Mn increased in cell walls and decreased in the soluble fraction, while proportion of Cd increased in the soluble fraction and decreased in cell walls. Together, these results indicated a dose dependent influence of Mn and Cd to pokeweed growth and metal accumulation but in different manners.

**Figure 2:**
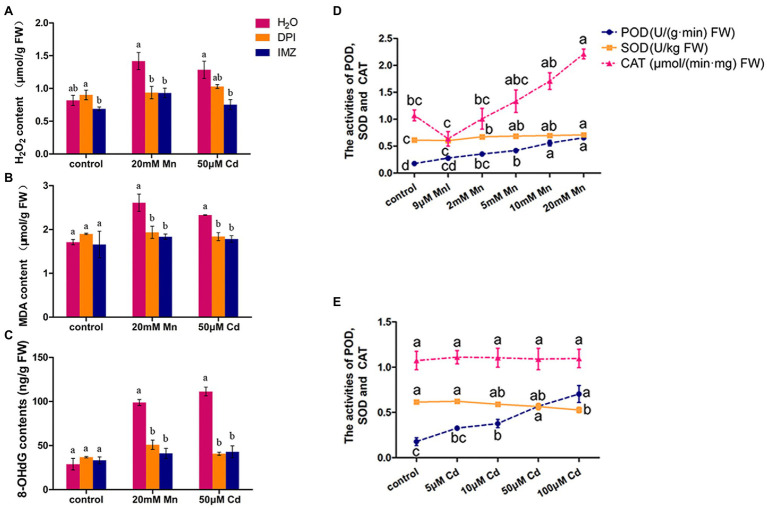
ROS accumulation and scavenging in response to excess Mn and Cd: **(A)** H_2_O_2_ content; **(B)** MDA content; and **(C)** 8-OHdG content. DPI, IMZ, and H_2_O refer to samples pretreated with NADPH oxidase inhibitors (DPI and IMZ) or H_2_O prior to heavy metal exposure. **(D,E)** The activities of POD, SOD, and CAT in leaves with Mn **(D)** and Cd **(E)** treatments. Data represent mean ± SD of six biological replicates per treatment. Letter annotations indicate significant differences according to Duncan’s multiple range test (*p* < 0.05).

We also detected the contents of H_2_O_2_, MDA, and 8-OHdG in pokeweed leaves to assess oxidative stress under treatments with excess Mn and Cd (20 mM Mn and 50 μM Cd). The result showed excess Mn and Cd treatments resulted in remarkable increases of these three substances, which suggested excess Mn and Cd triggered oxidative stress in pokeweed (Figure 2). When we pretreated a subset of seedlings with NADPH oxidase inhibitors (DPI and IMZ) prior to heavy metal exposure, the Mn-/Cd-induced MDA, H_2_O_2_, and 8-OHdG can be significantly inhibited (Figures 2A–C). These results suggested the disrupted NADPH oxidase could alleviate Mn-/Cd-induced oxidative stress. Then, we detected ROS-scavenging enzyme activities in response to increased Mn and Cd exposures (Figures 2D,E). The enhanced POD activities were detected with increasing Mn and Cd concentrations. However, the SOD activity increased with increasing Mn concentrations but reduced with increasing Cd concentrations. Besides, the enhanced CAT activity was only detected in treatments with increasing Mn concentrations. These results showed that ROS scavenging system exhibited different patterns under excess Mn and Cd in pokeweed.

### Different Expressions of Genes Involved in ROS-Scavenging System and DNA Methylation Responding to Mn and Cd Stress in Pokeweed

**Figure 3:**
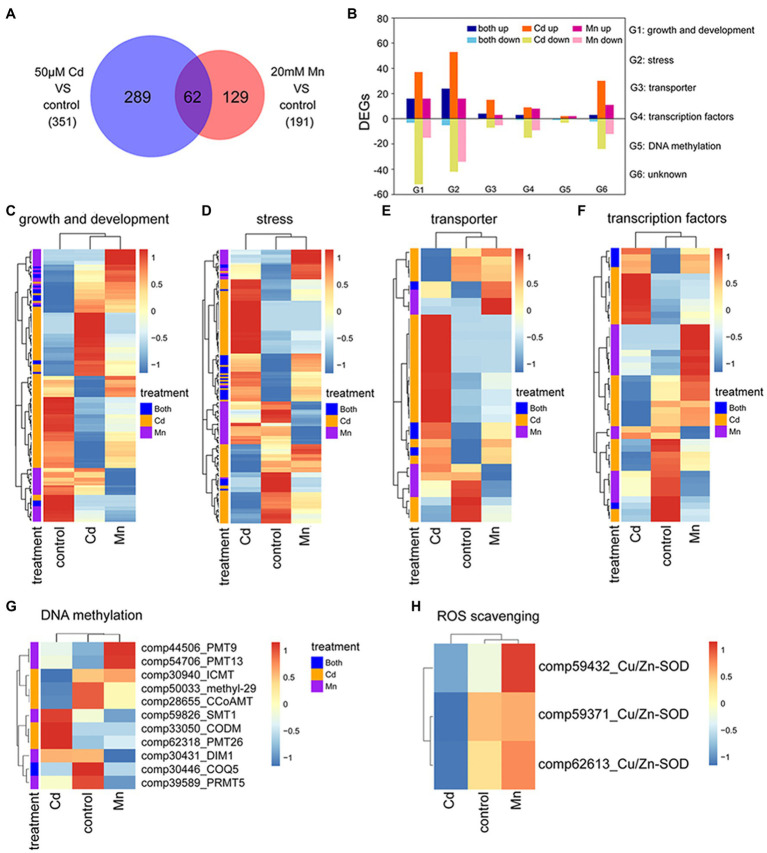
Transcriptomic analysis of seedlings subjected to 20-day Mn, Cd, and control treatments: **(A)** Venn diagram of DEGs under excess Mn and Cd; **(B)** number of DEGs assigned to different functions under excess Mn and Cd; **(C**–**G)** heatmaps of DEGs included in different functional groups (G1–G5 in panel **B**); **(H)** heatmaps of differential expressions of ROS scavenging-related genes.

We used transcriptome and expression profile analysis to detect DEGs among Mn and Cd treatments. The reliability of transcriptome analysis was verified by qRT-PCR results (Supplementary Table S2). Compared to the control treatment, we detected 191 DEGs in the 20 mM Mn treatment and 351 in the 50 μM Cd treatment (Figure 3A; Supplementary Tables S4, S5). Among these DEGs, 289 (82%) and 129 (68%) DEGs were specific for Mn and Cd treatments, respectively (Figure 3A). We used gene ontology (GO) classification to assess the functions of DEGs. Functions of all 480 DEGs (combination of DEGs for Mn and Cd treatments) were assigned to six categories, including growth and development (138%, 28.75%), stress (173%, 36.04%), transportation (34%, 7.08%), transcription factors (45%, 9.38%), DNA methylation (8%, 1.67%), and others (82%, 17.08%; Figure 3B). Interestingly, only a small percentage of DEGs in a category shared same regulation trend between Mn and Cd treatments (i.e., up or down simultaneously; Figure 3B), and most of DEGs in a category showed inconsistent trends (Figures 3C–H). Together, these results showed distinguished responses to Mn and Cd by pokeweed at transcriptional level.

Among these DEGs, eight methylation or demethylation related genes were found being differentially expressed under Mn or Cd stress (Figure 3G). Only one gene, 2-methoxy-6-polyprenyl-1,4-benzoquinol methylase (COQ5), was significantly down-regulated under both Mn and Cd stress. And the others showed significant up- or down-regulation for either Mn or Cd treatment. These results indicated that excess Mn or Cd might affect DNA methylation. Besides, differential expression of ROS-scavenging enzymes was observed under Mn and Cd stress. Expressions of three copper-zinc superoxide dismutases (Cu/Zn-SOD) were down-regulated under Cd stress, but up-regulated under Mn stress (Figure 3H), which confirmed the results of SOD activities.

**Figure 4:**
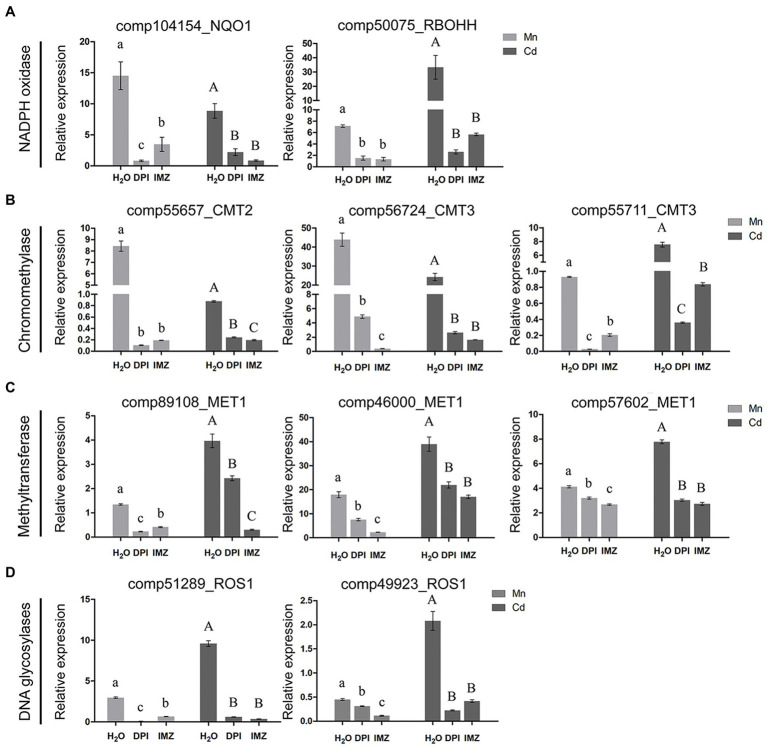
qRT-PCR analysis of **(A)** NADPH oxidase; **(B)** Chromomethylase; **(C)** Methyltransferase; and **(D)** DNA glycosylase homologous genes. The relative expression of alpha-3 and -5 tubulin served as an internal control. DPI, IMZ, and H_2_O refer to samples pretreated with NADPH oxidase inhibitors (DPI and IMZ) or H_2_O prior to heavy metal exposure. Data represent mean ± SD of three biological replicates. Letter annotations indicate significant differences according to Duncan’s multiple range test (*p* < 0.05).

Notably, we found an association between the expressions of DNA methylation related genes and the activity of Mn-/Cd-induced ROS (Figures 4B–D). Under excess Mn and Cd treatments, two NADPH oxidase homolog genes (comp104154_NQO1 and comp50075_RBOHH) were detected being significantly up-regulated. When we pretreated the pokeweed with two NADPH oxidase inhibitors (DPI and IMZ), such up-regulations were inhibited and ROS productions were further decreased (Figures 2A, 4A). Meanwhile, we investigated the expressions of eight DNA methylase or demethylase homolog genes (Figures 4B–D). The expressions of three chromomethylase genes (comp55657_CMT2, comp56724_CMT3, and comp55711_CMT3) were up-regulated under Mn and Cd treatments while pretreatment with DPI and IMZ inhibited increases of these gene expressions induced by excess Mn or Cd (Figure 4B). In the same way, expression of three methyltransferase genes (comp89108_MET1, comp46000_MET1, and comp57602_MET1) were up-regulated under excess Mn and Cd treatments, and pretreatment inhibited these Mn-/Cd-induced increases of gene expressions (Figure 4C). Similar results were also detected for two DNA glycosylases genes (Figure 4D). These results suggested that the up-regulated DNA methylase and demethylase homolog genes under excess Mn and Cd treatments can be suppressed to some extent when pretreated with NADPH oxidase inhibitors. Inhibition of NADPH oxidase activity led to a decrease in ROS contents which further affected DNA methylation regulations. Thus metal-induced ROS might play important roles in methylation variation of pokeweed in response to heavy metal stress.

### Dose Dependent Changes in DNA Methylation Under Mn and Cd Stress

**Figure 5:**
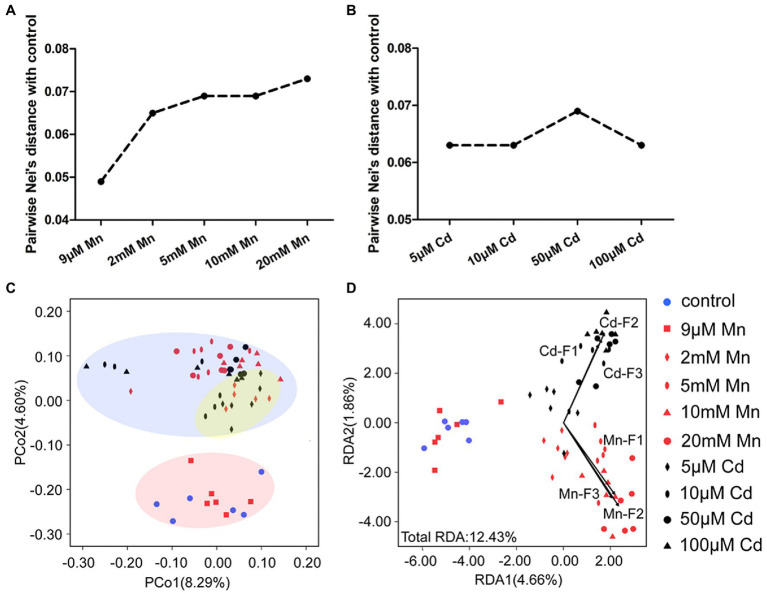
DNA methylation analysis based on MSAP profiling of pokeweed under a series of Mn or Cd treatments. **(A,B)** MSAP-based Nei’s distances between the control group and **(A)** Mn treatments and **(B)** Cd treatments. **(C)** Principal coordinates analysis (PCoA) of changes in DNA methylation in seedlings subjected to 20 days treatment of Mn and Cd. **(D)** Redundancy analyses (RDA) of changes in DNA methylation changes using Mn and Cd concentrations in cell walls (F1), organelles (F2), and the soluble fraction (F3) as explanatory variables under different treatments.

We used MSAP profiling to detect the detailed DNA methylation pattern of pokeweed under treatments with series of Mn and Cd concentrations (Figure 5). This analysis yielded 855 epigenetic loci, 823 of which were MSL loci. A clear dose dependent change of DNA methylation under Mn and Cd stress was detected. First, the pairwise Nei’s epigenetic distance between the control and Mn/Cd treatments mainly increased with increasing metal concentrations (Figures 5A,B). Furthermore, the PCoA pointed to three clusters of Mn and Cd treatments (Figure 5C): the “normal” cluster primarily represented the control and 9 μM Mn treatments; the “low degree of stress” cluster primarily referred to the 2 mM Mn and 5 μM Cd treatments; and the remaining Mn treatments and Cd treatments were assigned to the “excess” cluster. Finally, we used Mn and Cd content in the three cellular components (Figures 1G,H) as indicators of phenotype and analyzed their relationship with DNA methylation with RDA (Figure 5D). The contents of Mn and Cd in the three cellular components were significantly correlated with variations of methylated loci in pokeweed under a series of Mn and Cd concentrations, and the Mn and Cd contents could explain 12.43% variation of DNA methylation under Mn and Cd treatments (Figure 5D; Supplementary Table S6). Besides, we used MSAP variation to explain changes in DEGs expression. The variability described by the first two RDA axes retains 56.98% of DEG variability under 20 mM Mn and 50 μM Cd treatments, and no square was contained in the unconstrained axes (Supplementary Figure S2; Supplementary Table S7).

### Relationship Between DNA Methylation and the Content of ROS Under Mn and Cd Stress

**Figure 6:**
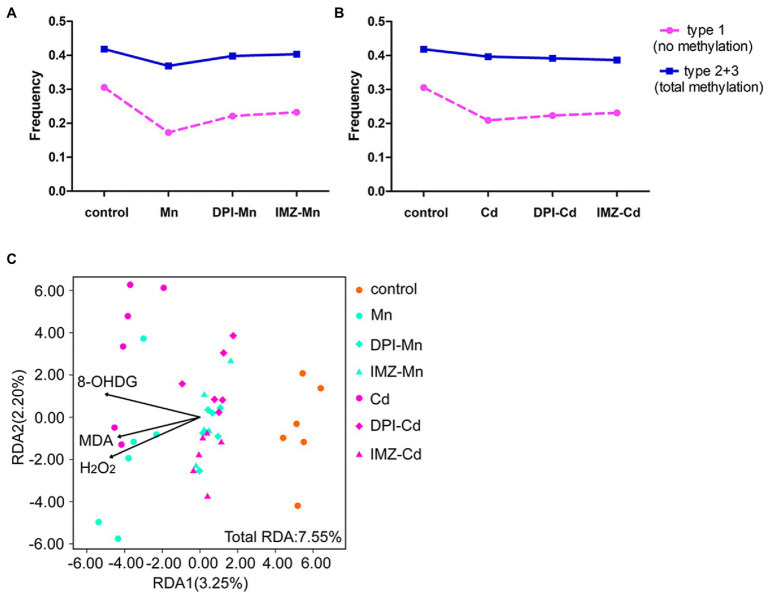
The changes of DNA methylation variation based on MSAP profiling of pokeweed under excess Mn/Cd treatments or pretreated with NADPH oxidase inhibitors. **(A,B)** Frequencies of the four methylation types in seedlings exposed to **(A)** Mn treatments and **(B)** Cd treatments. DPI-Mn/Cd, IMZ-Mn/Cd, and Mn/Cd refer to samples pretreated with NADPH oxidase inhibitors (DPI and IMZ) or H_2_O prior to heavy metal exposure. **(C)** Redundancy analysis (RDA) to evaluate the relative importance of oxidative stress biomarkers (MDA, H_2_O_2_, and 8-OHdG) as explanatory variables of DNA methylation variation among treatments.

To further assess the effects of ROS on DNA methylation, we compared the frequency of methylated loci (including non-methylated and total methylated type) between pretreated and non-pretreated samples with NADPH oxidase inhibitors using MSAP profiling (Figures 6A,B). We found that declines of frequency in non-methylated loci under Mn and Cd treatments were mediated partly by ROS, which were reduced if oxidative stress was alleviated by DPI or IMZ. Compared with control samples, the frequency of Type 1 (indicating no methylation) was decreased by 43.47% in non-pretreated samples in the 20 mM Mn treatment, as compared to a 27.63% decrease in DPI-treated samples and a 24.03% decrease in IMZ-treated samples (Figure 6A). Similarly, Type 1 decreased by 31.59% in non-pretreated samples in the 50 μM Cd treatment, as compared to reductions of 26.97% and 24.35% in the DPI- and IMZ-treated samples, respectively (Figure 6B). The results were consistent with the inhabitation of Mn- and Cd-induced increases of gene expressions involved in DNA methylase and demethylase (Figures 4B–D). We also analyzed sample-level relationships between oxidative stress biomarkers and DNA methylation with RDA, using the contents of MDA, H_2_O_2_, and 8-OHdG as explanatory variables, which showed MDA, H_2_O_2_, and 8-OHdG exerted significant influences on DNA methylation (Figure 6C; Supplementary Table S6).

**Figure 7:**
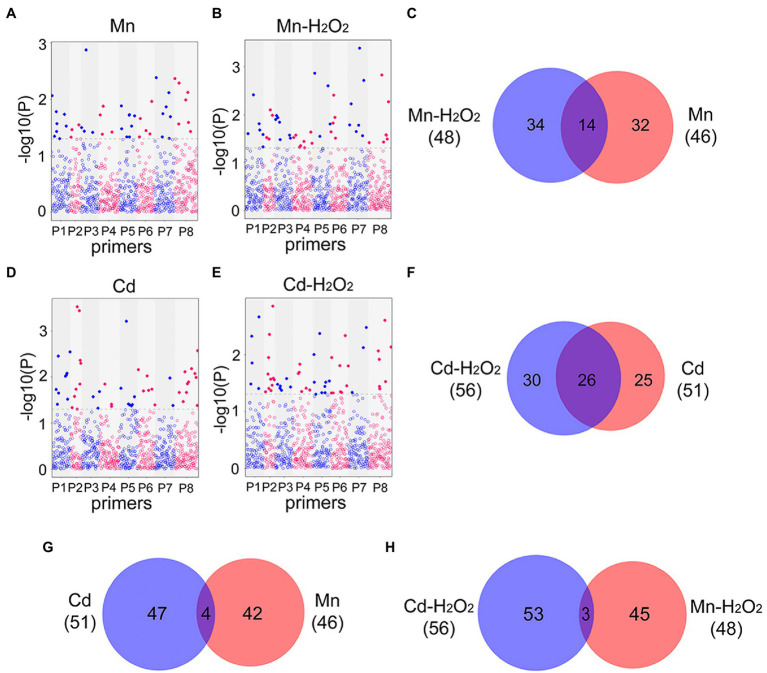
Manhattan plots of total methylated loci significantly associated with **(A)** leaf Mn content in Mn treatments (Mn); **(B)** leaf H_2_O_2_ content in Mn treatments (Mn-H_2_O_2_); **(D)** leaf Cd content in Cd treatments (Cd); and **(E)** leaf H_2_O_2_ content in Cd treatments (Cd-H_2_O_2_). The x-axis represents different loci amplified in eight MSAP primer pairs (Supplementary Table S1), and the y-axis is −log10 (value of *p*). Blue line indicates *p* < 0.05, and red line indicates *p* < 0.01. The Venn diagrams show the comparisons of heavy metal associated and H_2_O_2_ associated differentially methylated loci (DML, *p* < 0.05) detected in the four Manhattan plots. **(C)** Venn diagram of Mn and H_2_O_2_ associated DML in Mn treatment; **(F)** Venn diagram of Cd and H_2_O_2_ associated DML in Cd treatment; **(G)** Venn diagram of Cd and Mn associated DML in Cd and Mn treatments respectively; **(H)** Venn diagram of H_2_O_2_ associated DML in Cd and Mn treatments.

Next, we performed EWAS analysis to examine the relationship between methylated loci (including total and no methylated loci) and phenotype traits (the contents of Mn, Cd, and H_2_O_2_). For total methylated loci (type 2 + 3), 46 loci were associated with Mn content and 48 loci were associated with Mn induced H_2_O_2_ (Mn-H_2_O_2_) content in leaves under the Mn treatments (Figures 7A–C), whereas 51 loci were associated with Cd content and 56 loci were associated with H_2_O_2_ (Cd-H_2_O_2_) content under the Cd treatments (*p* < 0.05, Figures 7D–F). Among these phenotype-trait associated methylated loci (DML), we identified 14 (30%) DML shared among Mn and Mn-H_2_O_2_, and 26 (51%) shared among Cd and Cd-H_2_O_2_ (Figures 7C,F). By contrast, only four DML were shared between Mn and Cd, and three between Mn-H_2_O_2_ and Cd-H_2_O_2_ (Figures 7G,H). The results for non-methylated loci (type 1) followed the same trend as total methylated loci (Supplementary Figure S3). These results indicated Mn- and Cd-specific DML were induced by excess Mn and Cd, and confirmed that methylation resulting from excess Mn and Cd were at least partially mediated by ROS.

## Discussion

Although DNA methylation variation has been observed in many plant species in response to heavy metal stress, previous studies have focused mainly on changes in methylation levels (Dowen et al., 2012; Ou et al., 2012; Shi et al., 2017). However, multiple studies have shown that patterns in methylation levels vary by heavy metal type, and some studies have reported conflicting results (Yaish, 2013; Cong et al., 2019; Fan et al., 2020). This suggests that heavy metal-induced DNA methylation in plants is a relatively complicated process in need of further exploration. Here, we systematically analyzed the responses of pokeweed to a range of concentrations of Mn and Cd at phenotypic, transcriptional and DNA methylation levels. The variation pattern of methylated loci in pokeweed was significantly correlated with the content of heavy metals. A susceptible-concentration change of DNA methylation was detected in pokeweed under a series of Mn and Cd treatments (Figure 5). EWAS analysis showed that Mn- and Cd-specific DML were induced by excess Mn and Cd (Figure 7). Those above results indicating site-specific methylation patterns, rather than overall methylation levels, may be the key to response to different heavy metals. Moreover, pokeweed distinguished different heavy metals with metal-specific methylation sites, potentially in a dose-dependent manner.

In addition, we demonstrated that ROS play an important role in mediating heavy metal-induced changes in DNA methylation in plants. ROS are common products of heavy metal stress (Shahid et al., 2014), and their production is promoted by activated NADPH oxidase (Choudhury et al., 2017). Our results indicate that the content of H_2_O_2_, MDA, and 8-OHdG increased markedly in the Mn and Cd treatments, suggesting that heavy metal stress promotes accumulation of excess ROS and induces oxidative stress, resulting in effects such as lipid peroxidation and DNA damage. However, when samples were pretreated with NADPH oxidase inhibitors, the content of H_2_O_2_, MDA, and 8-OHdG were substantially lower than in non-pretreated samples (Figures 2A–C). The activity of methylase (CMT2, CMT3, and MET1) and demethylase (ROS1) also decreased markedly in samples pretreated with NADPH oxidase inhibitors, whereas non-methylated loci increased (Figures 4B,C). These results indicated that metal-induced ROS may affect the genome by inhibiting DNA methylation. Other studies have reported that ROS can result in increased DNA demethylation (Aina et al., 2004; Dowen et al., 2012; Yaish, 2013; Bernardo et al., 2017). One explanation of DNA methylation suppression by metal-induced oxidative stress is that ROS-induced 8-OHdG may interfere with the ability of DNA to function as a substrate for DNA methyltransferase, resulting in hypomethylation (Takumi et al., 2015; Shi et al., 2017). In our study, EWAS analysis indicated that a series of heavy metal-related DML also were H_2_O_2_-related DML, providing further evidence that changes in DNA methylation under Mn and Cd stress are likely affected in part by metal-induced ROS (Figure 7; Supplementary Figure S3). The hypomethylation under heavy metal stress reported in many other studies (Ding et al., 2014; Feng et al., 2016; Fan et al., 2020) may be partially attributable to metal-induced ROS, and ROS may play an important mediating role in the process of metal-induced DNA methylation in plants.

It is worth noting that, although excess Mn and Cd may cause ROS accumulation, ROS generation and scavenging pathways also exhibited changes. Other studies have reported that Mn has redox properties and may directly induce ROS production through Fenton and Haber–Weiss reactions (Schützendübel and Polle, 2002; Halliwell, 2006), whereas Cd, as a non-redox active metal, only stimulates ROS production indirectly *via* mechanisms such as the disruption of biomacromolecule activity (Halliwell, 2006; Shahid et al., 2014). Our study showed that the ROS-scavenging activities in leaves differed under Mn and Cd stress (Figures 2D,E, 3H). In addition, we observed that, although ROS can inhibit DNA methylation, there was scarce overlap between DML related to Mn-ROS and DML related to Cd-ROS (Figure 7H; Supplementary Figure S3H). It remains unclear whether different ROS production and scavenging systems lead to different ROS-induced patterns in DNA methylation.

It has been reported repeatedly that DNA methylation involves in gene expression regulation in plants (Harris et al., 2018; Zhang et al., 2018). For example, Feng et al. (2016) reported that Cd-responsive gene expression may be modified by DNA methylation in rice exposed to Cd. Our recent study of pokeweed also proved that DNA methylation variation induced by bacteria can regulate gene expression (Chen et al., 2022). Although transcriptome analysis of *P. americana* L. in response to Cd stress have been reported before (Chen et al., 2017), they did not pay attention to the effect of DNA methylation on gene expression and did not compare the difference of DEG under Mn and Cd stress. In our study, we found that under Mn and Cd stress, pokeweed employs various molecular mechanisms with respect to transcription, revealed by that a large number of DEGs differed between the Mn and Cd treatments (Figure 3). These DEGs mainly involved in growth and development, transcription factors, transportation and stress. In addition, our RDA analysis indicated that DNA methylation variation exerted significant influences on gene expression responding to heavy metal stress (Supplementary Figure S2; Supplementary Table S7). Combined with the finding that differential DNA methylations and phenotypes responded to Mn and Cd stress in pokeweed, our result showed that changes in methylation might result in transcriptional regulation and phenotypic variation in pokeweed under heavy metal stress, which was essential to the adaptive response of plants to heavy metal stress.

## Conclusion

**Figure 8:**
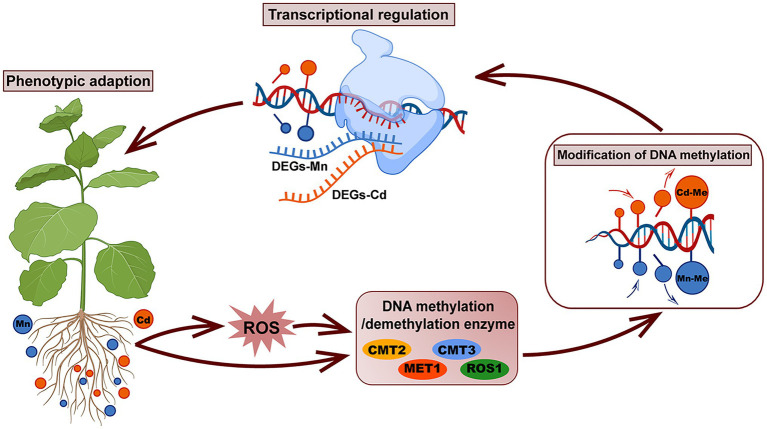
A schematic summary of the phenotypic adaption of pokeweed under Mn and Cd stress mediated by DNA methylation and transcriptional regulation. In pokeweed, DNA methylase and demethylase were differently expressed caused by Mn and Cd stress directly or mediated by Mn/Cd-induced ROS indirectly. The different expressions of DNA methylase and demethylase further triggered different modification of DNA methylation leading to Mn- and Cd-specific DNA methylated loci, which showed that partial changes in DNA methylation under Mn/Cd stress were mediated by Mn/Cd-induced ROS. The Mn- and Cd-specific DNA methylated loci further distinguished Mn and Cd with different transcriptional patterns and finally induced different phenotypes responses to Mn and Cd stress.

Our study demonstrated that pokeweed distinguished Mn and Cd with different subcellular distributions, ROS scavenging systems, and transcriptional and DNA methylation patterns (Figure 8). Heavy metal-induced DNA methylation was associated with gene expression and phenotypic variation, which suggested that DNA methylation was a rapid response strategy promoting pokeweed survival under heavy metal stress. In pokeweed, Mn and Cd induced different DNA methylation sites in a dose-dependent manner. The site-specific methylation patterns, rather than the overall methylation levels, might be the key for pokeweed to response to different heavy metals. Moreover, ROS played an important role in mediating heavy metal-induced changes in DNA methylation in pokeweed. Heavy metal-induced ROS may result in increased levels of demethylation, and a portion of the changes in DNA methylation under Mn and Cd stress may be regulated by the metal-induced ROS, suggesting that ROS were likely to act as intermediaries under heavy metal stress.

## Data Availability Statement

The datasets presented in this study can be found in online repositories. The names of the repository/repositories and accession number can be found at: https://www.ncbi.nlm.nih.gov/bioproject/?term=PRJNA623405.

## Author Contributions

MJ contributed to conceptualization, methodology, investigation, and writing—original draft preparation. HZ, MW, and YT contributed to investigation and data curation. YX and YC contributed to writing—reviewing and editing. ZS contributed to conceptualization, supervision, writing—reviewing and editing, and funding acquisition. CC contributed to conceptualization, supervision, writing—original draft preparation, writing—reviewing and editing, and funding acquisition. All authors contributed to the article and approved the submitted version.

## Funding

This work was supported by grants from the National Natural Science Foundation of China (31770404), the Fundamental Research Funds for the Central Universities (KYZ201739), the National Key Research and Development Program of China (2016YFD0800803), “China Agriculture Research System” China Postdoctoral Science Foundation (2016T90468), and the (CARS-10-B24). These funds received for open access publication fees.

## Conflict of Interest

The authors declare that the research was conducted in the absence of any commercial or financial relationships that could be construed as a potential conflict of interest.

## Publisher’s Note

All claims expressed in this article are solely those of the authors and do not necessarily represent those of their affiliated organizations, or those of the publisher, the editors and the reviewers. Any product that may be evaluated in this article, or claim that may be made by its manufacturer, is not guaranteed or endorsed by the publisher.

## Footnotes

^1^ http://www.biovenn.nl/index.php
