# Supplementary material for: Reactive Oxygen Species Partly Mediate DNA Methylation in Responses to Different Heavy Metals in Pokeweed
Source: Front Plant Sci. 2022 Apr 7;13:845108. doi: 10.3389/fpls.2022.845108 (PMC9021841; doi:10.3389/fpls.2022.845108)
Supplement: Supplementary file 1 [file Table_1.docx]

Supplementary Material

# Supplementary Materials and Methods

# 2.2 Phenotypic analysis

# Photosynthetic pigment content and gas exchange

# Photosynthetic pigment content was determined using the method described by Knudson et al. (1977). Photosynthetic pigments were extracted by soaking a 0.10 g leaf sample in 10 mL of 95% ethanol and measuring absorption of the extracts at 470 nm, 649 nm, and 665 nm using spectrophotometry (UV-2450, Shimadzu, Kyoto, Japan). Chlorophyll a, chlorophyll b, and total carotenoid content were calculated using equations from Lichtenthaler and Wellburn (1983), and photosynthetic gas exchange parameters were measured according to Chen et al. (2017). Photosynthetic gas exchange parameters including net photosynthetic rate (Pn), intercellular CO_2_ concentration (Ci), stomatal conductance (Gs), and transpiration rate (Tr) were measured between 08:30 and 11:30 using a Li-6400 portable photosynthesis system (LI-COR Biosciences, Lincoln, NE, USA) equipped with a LED light source.

# Mn and Cd accumulation in cellular components

# We used a slight modification of the methods described in Weigel and Jäger (1980) to determine the distribution of accumulated Cd and Mn in leaf cells. We added a 1.0 g fresh leaf sample to 10 mL of pre-chilled extractant [0.25 M sucrose, 50 mM Tris-HCI buffer (pH 7.5), l mM erythritol, and 5.0 mM ascorbic acid] and homogenized the mixture in an ice bath. We then centrifuged the homogenate at 300× g for 5 minutes, and then collected the pellet containing cell wall components (F1). The supernatant was centrifuged again at 20,000× g for 25 minutes; the pellet containing organelle components (F2) was collected, and the remaining supernatant comprised the soluble fraction (F3). Collections of F1, F2, and F3 were digested with HNO_3_-HClO_4_ (87:13, v:v; Zhao et al., 1994), and concentrations of Cd or Mn were measured using inductively coupled plasma optical emission spectrometry (ICP-OES; Agilent 710 Series ICP Optical Emission Spectrometer, Agilent Technologies, Santa Clara, CA, USA).

# Hydrogen peroxide (H_2_O_2_), malondialdehyde (MDA), and 8-hydroxy-2'-deoxyguanosine (8-OHdG) measurements

# The content of H2O2, a common ROS, was measured using the method described by Velikova et al. (2000). A 0.2 g fresh leaf sample was homogenized in 2 mL of 0.1 % (w/v) trichloroacetic acid (TCA) and centrifuged at 12,000× g for 15 minutes. We then added 0.5 mL of the supernatant to 1 mL of 1 M KI and 0.5 mL of 10 mM potassium phosphate buffer (pH 7.0). We measured H_2_O_2_ content by assessing the absorbance of titanium-peroxide complex at 390 nm. Absorbance values were calibrated to a standard curve generated using a known concentration of H_2_O_2_.

# Lipid peroxidation was measured by assessing the reaction of MDA to thiobarbituric acid (TBA) using the method described by Chen et al. (2017) with minor modifications. A 0.5 g fresh leaf sample was homogenized in 5 mL of 10% (w/v) trichloroacetic acid (TCA) and centrifuged at 20,000× g for 25 minutes. We then added 2 mL of supernatant to 2 mL of 0.67% (w/v) TBA containing 10% TCA. The mixture was heated at 95 °C for 30 minutes and then cooled rapidly in an ice bath. Following centrifugation at 12,000× g for 10 minutes, the absorbance of the supernatant was recorded at 450 nm, 532 nm, and 600 nm. The concentration of MDA was calculated as follows: MDA concentration (µmol/g FW) = [6.45 × (A532 − A600) − 0.56 × A450] × v/w, where v represents the volume of the extraction solution.

# Content of 8-OHdG was used as a proxy for DNA damage and measured using the method described by Shi et al. (2017). A 1.0 g leaf sample was collected and ground with 4 mL of 10 mM phosphate buffer solution (pH 7.2–7.4). The solution was then incubated using a Plant 8-OHdG ELISA Kit (Omnimabs, Alhambra, CA, USA), and the content of 8-OHdG was assessed using Multimode Plate Readers (TECAN Infinite 200, Männedorf, Switzerland) at 450 nm.

# Assays of the antioxidant enzyme activities

# Enzymatic antioxidant activity of pokeweed was investigated by carrying out superoxide dismutase (SOD), peroxidase (POD) and catalase (CAT) activity assays. The method of enzyme extraction was followed Wang et al. (2019). Fresh leaf segments (0.5 g) were homogenized in 5 mL pre-cooled 100 mM potassium phosphate buffer (pH 7.0) containing 1 mM Ethylenediaminetetraacetic acid disodium salt (EDTA-Na_2_) and 1% polyvinylpyrrolidone (PVP). The homogenate was centrifuged at 12,000× g for 20 min 4 °C and the supernatant used for the enzyme assays below.

# SOD activity was assayed by monitoring the photochemical reduction inhibition of nitroblue tetrazole (NBT) following the method of Giannopolitis and Ries (1977). One unit of SOD activity was defined as the amount of enzyme required to inhibit NBT reduction by 50% as monitored at 560 nm. POD activity was measured according to the method of Kenten and Mann (1954）and modified by Chen et al. (2017). The growing absorbance at 470 nm was monitored for 1 min as guaiacol was oxidized and the extinction coefficient was 26.6 mM^-1^ cm^-1^. CAT activity was determined by monitoring reduce of H_2_O_2_ at 240 nm within 3 minutes (Aebi, 1984).

# References

# Aebi, H., 1984. Catalase in vitro. Methods Enzymol. 105, 121-126. https://doi.org/10.1016/S0076-6879(84)05016-3.

# Chen, Q., Zhao, X., Lei, D., Hu, S., Shen, Z., Shen, W., & Xu, X., 2017. Hydrogen-rich water pretreatment alters photosynthetic gas exchange, chlorophyll fluorescence, and antioxidant activities in heat-stressed cucumber leaves. Plant Growth Regul. 83, 69-82. https://doi.org/10.1007/s10725-017-0284-1.

# Giannopolitis, C. N., & Ries, S. K., 1977. Superoxide Dismutase: I. Occurrence in higher plants. Plant Physiol. 59, 309-314. https://doi.org/10.1104/pp.59.2.309.

# Kenten, R. H. & Mann, P. J. G., 1954. A simple method for the preparation of horseradish peroxidase. Biochem. J. 57, 347-348. https://doi.org/10.1042/bj0570347.

# Knudson, L. L., Tibbitts, T. W., & Edwards, G. E., 1977. Measurement of ozone injury by determination of leaf chlorophyll concentration. Plant Physiol. 60, 606-608. https://doi.org/10.1104/pp.60.4.606.

# Lichtenthaler, H. K., & Wellburn, A. R., 1983. Determinations of total carotenoids and chlorophylls a and b of leaf extracts in different solvents. Biochem. Soc. Trans. 11, 591-592. https://doi.org/10.1042/bst0110591.

# Shi, D., Zhuang, K., Xia, Y., Zhu, C., Chen, C., Hu, Z., & Shen, Z., 2017. Hydrilla verticillata employs two different ways to affect DNA methylation under excess copper stress. Aquat. Toxicol. 193, 97-104. https://doi.org/10.1016/j.aquatox.2017.10.007.

# Velikova, V., Yordanov, I., & Edreva, A., 2000. Oxidative stress and some antioxidant systems in acid rain-treated bean plants: Protective role of exogenous polyamines. Plant Sci. 151, 59-66. https://doi.org/10.1016/s0168-9452(99)00197-1.

# Wang, Y., Yang R., Zheng J., Shen Z., & Xu X., 2019. Exogenous foliar application of fulvic acid alleviate cadmium toxicity in lettuce (Lactuca sativa L.). Ecotoxicol. Environ. Saf. 167, 10-19. https://doi.org/10.1016/j.ecoenv.2018.08.064.

# Weigel, H. J., & Jäger, H. J., 1980. Subcellular distribution and chemical form of cadmium in bean plants. Plant Physiol. 65, 480-482. https://doi.org/10.1104/pp.65.3.480.

# Zhao, F., McGrath, S. P., & Crosland, A. R., 1994. Comparison of three wet digestion methods for the determination of plant sulphur by inductively coupled plasma-atomic emission spectroscopy (ICP-AES). Commun. Soil Sci. Plant Anal. 25, 407-418. https://doi.org/10.1080/00103629409369047.

# 2. Supplementary Figures and Tables

## Supplementary Figures


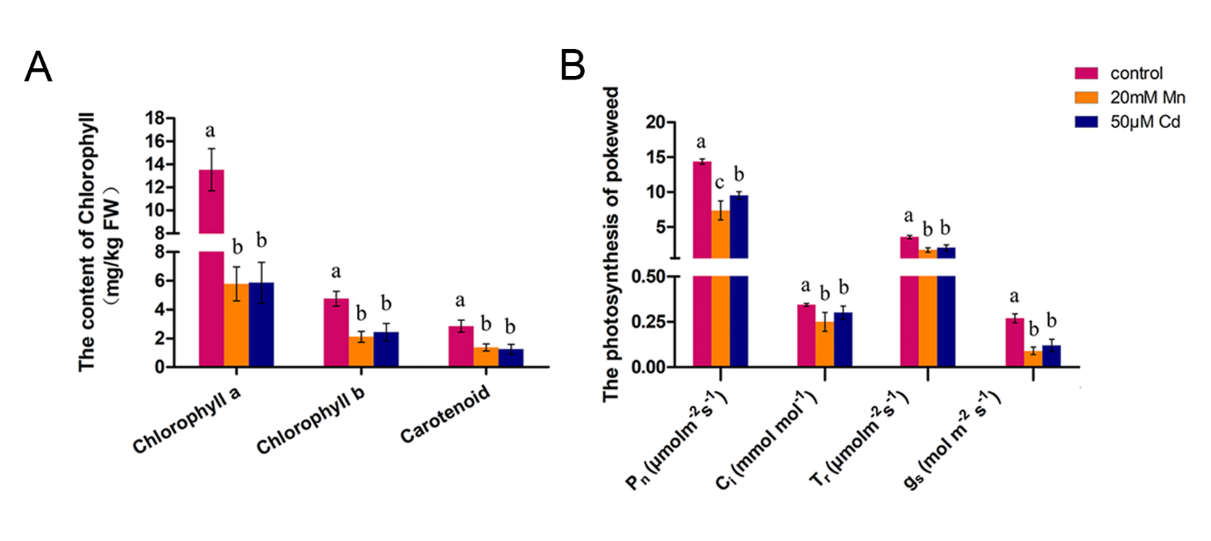


**Fig. S1.** Suppressions of photosynthesis in seedlings by excess Mn and Cd. (A) chlorophyll content; (B) parameters of photosynthetic gas exchange. Data represent mean ± SD of three biological replicates per treatment. Letter annotations indicate significant differences according to Duncan's multiple range test (*p* < 0.05).


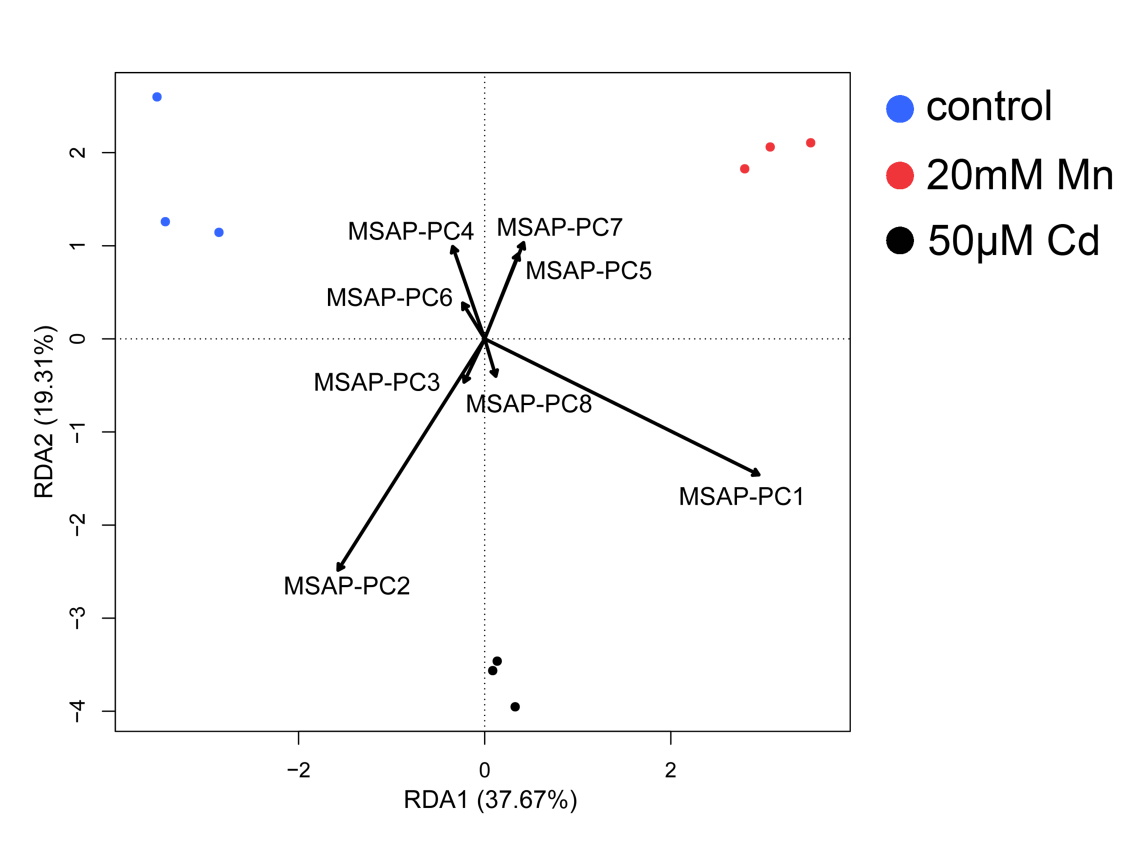


**Fig. S2.** Redundancy analyses (RDA) of changes in DEGs expression using the first eight PCA axes of MSAP profile (MSAP-PC1, MSAP-PC2, MSAP-PC3, MSAP-PC4, MSAP-PC5, MSAP-PC6, MSAP-PC7, MSAP-PC8) as explanatory variables under 20 mM Mn and 50 µM Cd treatments.


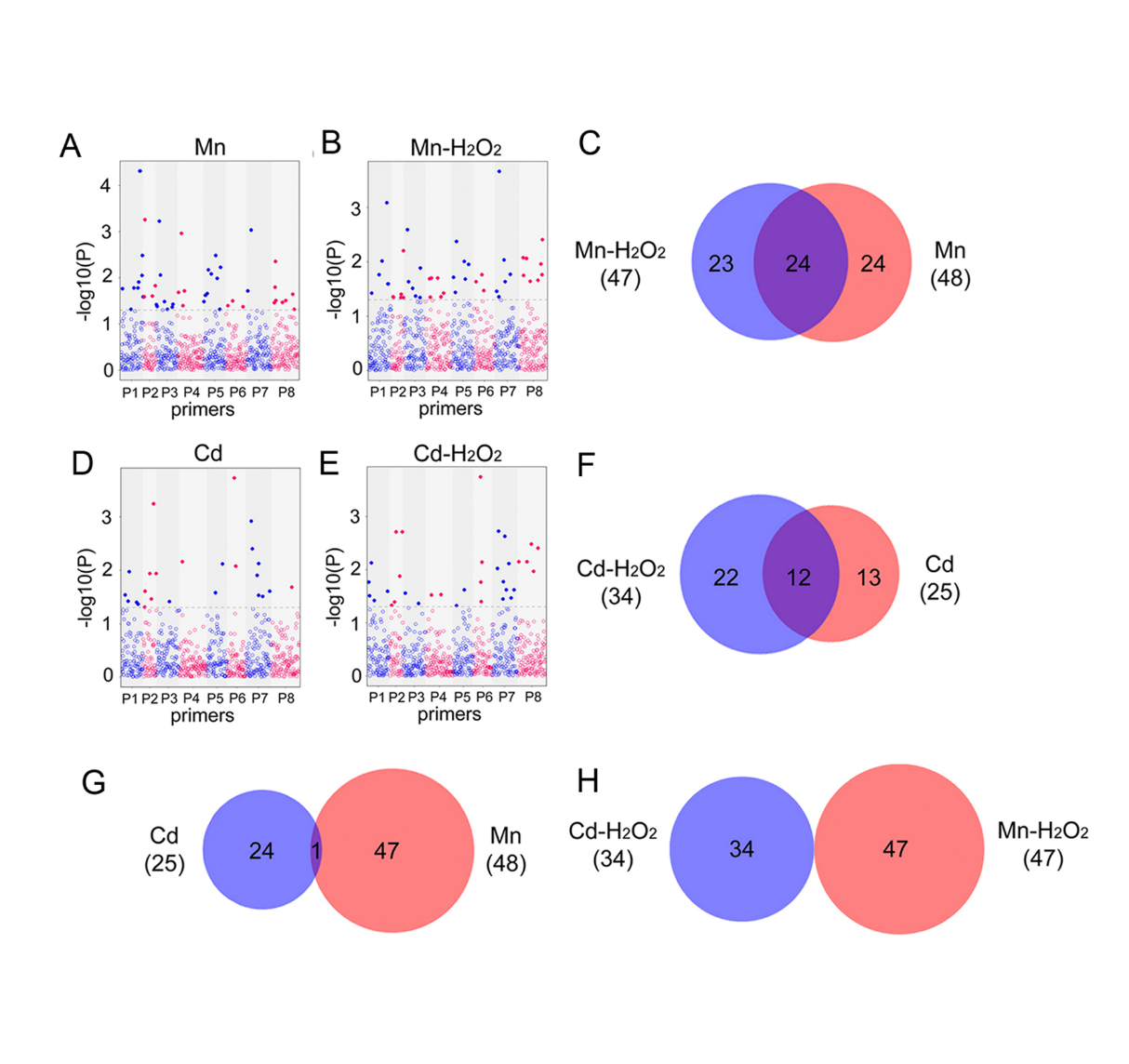


**Fig. S3.** Manhattan plots of non-methylated loci significantly associated with (A) leaf Mn content in Mn treatments (Mn); (B) leaf H_2_O_2_ content in Mn treatments (Mn-H_2_O_2_); (D) leaf Cd content in Cd treatments (Cd); and (E) leaf H_2_O_2_ content in Cd treatments (Cd-H_2_O_2_). The x-axis represents different loci amplified in eight MSAP primer pairs (Table S1), and the y-axis is −log10 (*p*-value). Blue line indicates *p* < 0.05, and red line indicates *p* < 0.01. The venn diagrams show the comparisons of heavy metal associated and H_2_O_2_ associated differentially methylated loci (DML, *p* < 0.05) detected in the four manhattan plots. (C) venn diagram of Mn and H_2_O_2_ associated DML in Mn treatment; (F) venn diagram of Cd and H_2_O_2_ associated DML in Cd treatment; (G) venn diagram of Cd and Mn associated DML in Cd and Mn treatments respectively; (H) venn diagram of H_2_O_2_ associated DML in Cd and Mn treatments.

## Supplementary Tables

**Table S1.** The qRT-PCR result of 21 random selected DEGs.

| Treatment | Unigene ID | Description | Log2 (fold-change) | |
| --- | --- | --- | --- | --- |
|  |  |  | DEGs | qRT-PCR |
| Cd | comp39805 | Eukaryotic translation initiation factor 3 subunit K isoform 1 | -8.78 | -6.46±0.22 |
|  | comp44377 | ATP synthase subunit O, mitochondrial | -4.97 | -4.00±0.30 |
|  | comp47915 | Zinc finger protein, putative | -9.68 | -4.77±0.11 |
|  | comp54759 | Mitochondrial-processing peptidase subunit alpha | -7.29 | -4.22±0.17 |
|  | comp9239 | Vesicle-associated membrane protein 727 | 9.02 | 5.55±0.35 |
|  | comp14096 | Benzyl alcohol O-benzoyltransferase-like | 7.77 | 3.38±0.17 |
|  | comp17966 | Glucan endo-1,3-beta-D-glucosidase | 10.21 | 6.70±0.23 |
|  | comp30949 | Phosphoenolpyruvate carboxylase, partial | 11.32 | 4.98±0.25 |
|  | comp52237 | Protein disulfide-isomerase-like isoform X1 | 12.36 | 6.36±0.14 |
|  | comp59432 | Cu/Zn superoxide dismutase | 10.1 | 6.30±0.32 |
|  | comp63608 | DNA-directed RNA polymerases I, II, and III subunit RPABC1-like | 10.08 | 6.77±0.23 |
| Mn | comp15708 | L-ascorbate peroxidase 6 | -10.16 | -6.29±0.093 |
|  | comp30446 | 2-methoxy-6-polyprenyl-1,4-benzoquinol methylase | -9.27 | -3.77±0..22 |
|  | comp83980 | Probable ATP-dependent RNA helicase YTHDC2-like | -9.12 | -4.93±0.26 |
|  | comp84817 | Proline transporter 1-like | -8.17 | -3.81±0.35 |
|  | comp7429 | DNA binding protein | 10.17 | 5.10±0.20 |
|  | comp27106 | Pheophorbide a oxygenase, chloroplastic-like | 11.03 | 5.92±0.32 |
|  | comp30829 | Vacuolar iron transporter 1-like | 8.5 | 5.83±0.27 |
|  | comp36781 | Transcription factor bHLH49 | 8.37 | 6.91±0.22 |
|  | comp43077 | Beta carbonic anhydrase 2 | 9.01 | 4.98±0.25 |
|  | comp59352 | Heavy metal-associated isoprenylated plant protein 26-like | 9.74 | 5.47±0.25 |

**Table S2.** The primer pairs used for qRT-PCR.

| gene ID | Primer | primer sequence**（5'-3'）** |
| --- | --- | --- |
| comp39805 | Forward primer | AAGAAGCAGCCAAGAACCGC |
|  | Reverse primer | ATGTTGAAAGCCTCCGAAAGAA |
| comp44377 | Forward primer | TCGTAAAGACGCTTCTCAACCC |
|  | Reverse primer | GGAACCTCCAAACATAGCAACAG |
| comp47915 | Forward primer | GCATAGCCTTTGTGCTTTGC |
|  | Reverse primer | CCTTCTTACCCTCTGGTTACGC |
| comp54759 | Forward primer | CCGTGAAGGCAGCAGGTCT |
|  | Reverse primer | TGGCAAAGGTGGCAGGATAG |
| comp9239 | Forward primer | CCTCCAATTTCGGGTTCG |
|  | Reverse primer | CGCCTCGTTGCTATCACTCA |
| comp14096 | Forward primer | TTCCTTACTATCCCTTCGCCG |
|  | Reverse primer | CAGAAGACGCTGGGACATCAAA |
| comp17966 | Forward primer | TTCTGAAAACTAATGGGTGTCCTC |
|  | Reverse primer | CTGGCTAACGCCGCATAAAC |
| comp30949 | Forward primer | CCGCCCATTCCTCCTAAGC |
|  | Reverse primer | GCAGAGATTCAATTCCGCCAC |
| comp52237 | Forward primer | GTCCCACTCCTCATCGTCCA |
|  | Reverse primer | CACCTTCCCTTCACTGTAGTCCTT |
| comp59432 | Forward primer | CCGCCACTTCCCATTATCAC |
|  | Reverse primer | TTTCCCTTGAGGACTGCGAC |
| comp63608 | Forward primer | AGAACTGGGAAGATGCCTGAG |
|  | Reverse primer | GGTGGAGACACGGAGACGAT |
| comp15708 | Forward primer | TCCTATTTCTTTCTGCCACTTTACC |
|  | Reverse primer | ACAACACCTTCCTTATCTCACCCT |
| comp30446 | Forward primer | GCGAGCACTTGCGAGG |
|  | Reverse primer | GCAGTCCAGCACTCATCAAA |
| comp83980 | Forward primer | GCTTGGACTCGGAGGATGG |
|  | Reverse primer | TTCTTTTGCTCAACAGATGGGAC |
| comp84817 | Forward primer | TTTGCCATTGTCGTGCCC |
|  | Reverse primer | CGAAGCCATTGAACACCTGA |
| comp7429 | Forward primer | GGAAGCCCGTCAATCAGC |
|  | Reverse primer | CCTCAGTCAATCTCCCAAACAA |
| comp27106 | Forward primer | GAGAGAGCCAGTGTTACCCAA |
|  | Reverse primer | CCTGTCCCTCCTTCCTGTG |
| comp30829 | Forward primer | TATTGTCGGAGTATGGGATAGAGG |
|  | Reverse primer | ATGTAGGCGATAGCGATTGTGA |
| comp36781 | Forward primer | GCTTGTCCCAGTTCCCTTCT |
|  | Reverse primer | TCTTCTGTGGCTGCCTAACG |
| comp43077 | Forward primer | ATGTGAGCCAGCAAGGTCCA |
|  | Reverse primer | CGTTTTCTTCACTACTGCTTCCC |
| comp59352 | Forward primer | CCACAAACGACGCAAACAGC |
|  | Reverse primer | GCACTCCCTTCATTCCTTCCA |
| comp104154_NQO1 | Forward primer | GTTGCCCTCGTTGTCGAACTT |
|  | Reverse primer | AGTACATCGACATGGCATCGT |
| comp50075_RBOHH | Forward primer | CCTGGTATGCTGTTAACCAGA |
|  | Reverse primer | GCTTCCAGTGTGTAGAAGAAC |
| comp55657_CMT2 | Forward primer | CGGATAACAGAAGAGGACGATGA |
|  | Reverse primer | CACAAACAACAGAGGTAAGGAGGA |
| comp56724_CMT3 | Forward primer | CAGTTTCATCCCACCATAGTCTTCC |
|  | Reverse primer | TCTACCAGGAGTTCGTGTTCGTC |
| comp55711_CMT3 | Forward primer | ATCTACCAGGAGTTCGTGTTCGTC |
|  | Reverse primer | AGTTTCATCCCACCATAGTCTTCC |
| comp46000_MET1 | Forward primer | TTGTAACCGAAGGCAAGTCTGA |
|  | Reverse primer | GGGATGAACTACCAAGCACGAAT |
| comp57602_MET1 | Forward primer | CATTACAGCCGTAGATTTGCCTCC |
|  | Reverse primer | ATCCACCTCAGCCAACACTCCC |
| comp89108_MET1 | Forward primer | GCTGCTAATGAAAGGGAGGTGT |
|  | Reverse primer | ACCGCACAATCCATTTCCAAC |
| comp51289_ROS1 | Forward primer | CCATAGGCAGTGGCTTGATAGG |
|  | Reverse primer | GTCTCGACCTTGAATGGTTACGT |
| comp49923_ROS1 | Forward primer | CAGTGAGGAATGCACCCGTAG |
|  | Reverse primer | TCGACACTCTTCAAGCCAAGC |

**Table S3.** Primer pairs used for MSAP selective amplifications.

| Primers/adapters | Oligonucleotide sequences (5′–3′) |
| --- | --- |
| EcoR I adapters |  |
| E1 | CTC GTA GAC TGC GTA CC |
| E2 | AAT TGG TAC GCA GTC TAC |
| Hpa II/Msp I adapters |  |
| HM1 | GAT CAT GAG TCC TGC T |
| HM2 | CGA GCA GGA CTC ATG A |
| Preselective primers |  |
| E01 | GAC TGC GTA CCA ATT CA |
| HM0 | ATC ATG AGT CCT GCT CGG |
| Selective primers |  |
| E14 | GAC TGC GTA CCA ATT CAAG |
| E31 | GAC TGC GTA CCA ATT CACA |
| E33 | GAC TGC GTA CCA ATT CACC |
| E34 | GAC TGC GTA CCA ATT CACG |
| HMTGC | CAT GAG TCC TGC TCG GTGC |
| HMTCA | CAT GAG TCC TGC TCG GTCA |
| HMTCCA | CAT GAG TCC TGC TCG GTCCA |
| HMTCGA | CAT GAG TCC TGC TCG GTCGA |

Note: P1 (E14*HMTGC)/P2 (E31*HMTCCA)/P3 (E33*HMTCGA)/P4 (E14*HMTCA)/P5 (E34*HMTCGA)/P6 (E33*HMTCCA)/P7 (E14*HMTCCA)/P8 (E14*HMTCGA).

**Table S4.** Information of 191 DEGs in pokeweed under 20mM Mn treatment.

| Gene_id | Control fpkm | Mn fpkm | FDR | log2FC | NR_Description |
| --- | --- | --- | --- | --- | --- |
| comp10179_c0 | 0.00 | 7.71 | 4.60E-02 | 9.02 | hypothetical protein POPTR_0015s10940g [Populus trichocarpa] |
| comp10488_c0 | 0.02 | 8.94 | 1.46E-02 | 9.82 | hypothetical protein PRUPE_ppa008539mg [Prunus persica] |
| comp11132_c0 | 0.00 | 11.10 | 2.99E-02 | 8.25 | PREDICTED: gibberellin-regulated protein 4-like [Vitis vinifera] |
| comp11728_c0 | 0.00 | 13.34 | 2.01E-02 | 9.61 | PREDICTED: 5'-adenylylsulfate reductase-like 4 isoform 1 [Vitis vinifera] |
| comp12226_c0 | 4.17 | 0.00 | 2.37E-02 | -8.42 | glucosyltransferase like family protein [Populus trichocarpa] |
| comp12436_c0 | 0.00 | 6.17 | 4.76E-02 | 9.28 | PREDICTED: synaptotagmin-5-like [Cucumis melo] |
| comp12730_c0 | 0.00 | 7.40 | 3.21E-02 | 8.44 | _ |
| comp12871_c0 | 0.00 | 4.84 | 4.27E-02 | 7.90 | conserved hypothetical protein [Ricinus communis] |
| comp13643_c0 | 0.00 | 5.28 | 2.86E-02 | 8.34 | mads box protein, putative [Ricinus communis] |
| comp14540_c0 | 0.00 | 3.99 | 2.17E-02 | 9.56 | PREDICTED: nuclear pore complex protein Nup98-Nup96 [Vitis vinifera] |
| comp15211_c0 | 0.00 | 5.16 | 5.27E-03 | 9.20 | aspartyl protease family protein [Populus trichocarpa] |
| comp15556_c0 | 6.87 | 0.00 | 2.86E-02 | -8.23 | PREDICTED: uncharacterized protein LOC101509344 [Cicer arietinum] |
| comp15708_c0 | 15.70 | 0.00 | 2.07E-04 | -10.16 | PREDICTED: putative L-ascorbate peroxidase 6 [Vitis vinifera] |
| comp15993_c0 | 0.00 | 11.29 | 1.95E-03 | 9.53 | PREDICTED: cytochrome P450 86A8-like [Glycine max] |
| comp15997_c0 | 0.00 | 8.25 | 2.04E-02 | 8.49 | PREDICTED: uncharacterized protein LOC101509344 [Cicer arietinum] |
| comp17003_c0 | 4.60 | 0.00 | 1.63E-02 | -9.69 | hypothetical protein PRUPE_ppa005569mg [Prunus persica] |
| comp19386_c0 | 15.31 | 0.00 | 6.72E-03 | -10.18 | PREDICTED: 5'-adenylylsulfate reductase-like 5-like [Citrus sinensis] |
| comp22397_c0 | 19.91 | 0.00 | 5.94E-12 | -11.92 | hypothetical protein JCGZ_18897 [Jatropha curcas] |
| comp23417_c0 | 0.00 | 11.48 | 3.25E-02 | 8.14 | _ |
| comp23572_c0 | 0.00 | 11.20 | 2.75E-03 | 9.44 | hypothetical protein [Beta vulgaris] |
| comp24422_c0 | 0.00 | 8.64 | 7.90E-03 | 10.14 | PREDICTED: scarecrow-like protein 8-like [Vitis vinifera] |
| comp25052_c0 | 7.25 | 0.00 | 4.10E-02 | -9.16 | PREDICTED: ATP-dependent Clp protease ATP-binding subunit ClpX-like [Vitis vinifera] |
| comp25340_c0 | 12.96 | 0.00 | 6.44E-05 | -10.34 | hypothetical protein EUGRSUZ_F04472 [Eucalyptus grandis] |
| comp25974_c0 | 0.00 | 15.50 | 1.89E-03 | 10.57 | PREDICTED: putative clathrin assembly protein At5g35200 [Prunus mume] |
| comp27039_c0 | 0.00 | 10.05 | 3.34E-04 | 10.01 | High mobility group family isoform 1 [Theobroma cacao] |
| comp27106_c0 | 0.00 | 14.55 | 3.57E-04 | 11.03 | PREDICTED: pheophorbide a oxygenase, chloroplastic-like [Fragaria vesca subsp. vesca] |
| comp27257_c0 | 17.41 | 0.00 | 6.03E-03 | -10.35 | PREDICTED: E3 ubiquitin-protein ligase RING1-like isoform 1 [Fragaria vesca subsp. vesca] |
| comp27456_c0 | 0.02 | 27.88 | 5.55E-03 | 11.55 | PREDICTED: acetyl-coenzyme A carboxylase carboxyl transferase subunit alpha, chloroplastic-like isoform X1 [Citrus sinensis] |
| comp27624_c0 | 0.00 | 9.98 | 1.83E-03 | 10.57 | sugar transporter, putative [Ricinus communis] |
| comp27629_c0 | 7.22 | 0.00 | 3.21E-02 | -8.11 | unnamed protein product [Vitis vinifera] |
| comp27746_c0 | 14.52 | 0.00 | 2.57E-02 | -9.62 | PREDICTED: MLP-like protein 28 [Vitis vinifera] |
| comp27762_c0 | 0.00 | 20.22 | 1.15E-08 | 11.30 | hypothetical protein JCGZ_01491 [Jatropha curcas] |
| comp27802_c0 | 0.00 | 21.62 | 4.63E-03 | 11.48 | PREDICTED: secologanin synthase-like [Cicer arietinum] |
| comp28150_c0 | 14.88 | 0.00 | 1.89E-02 | -8.60 | _ |
| comp28168_c0 | 0.00 | 8.37 | 1.55E-02 | 9.78 | hypothetical protein VITISV_027256 [Vitis vinifera] |
| comp28266_c0 | 0.00 | 11.16 | 3.34E-04 | 10.25 | PREDICTED: protein grpE [Vitis unnamed protein product [Vitis vinifera] |
| comp28315_c0 | 0.00 | 15.00 | 6.44E-05 | 10.31 | mitogen-activated protein kinase 2 [Glycine max] |
| comp28547_c0 | 0.00 | 14.65 | 1.17E-02 | 9.90 | hypothetical protein PRUPE_ppa009615mg [Prunus persica] |
| comp28555_c0 | 8.66 | 0.00 | 3.04E-02 | -10.43 | PREDICTED: uncharacterized protein LOC100241465 [Vitis vinifera] |
| comp28669_c0 | 34.42 | 0.00 | 1.50E-04 | -11.27 | hypothetical protein L484_005867 [Morus notabilis] |
| comp28708_c0 | 0.00 | 4.17 | 4.56E-02 | 9.03 | _ |
| comp28727_c0 | 0.00 | 12.16 | 2.70E-03 | 10.46 | PREDICTED: glycolipid transfer protein-like isoform X1 [Citrus sinensis] |
| comp28732_c0 | 0.00 | 9.29 | 7.14E-03 | 9.02 | PREDICTED: high mobility group B protein 7-like [Vitis vinifera] |
| comp28739_c0 | 0.00 | 8.14 | 2.57E-02 | 8.36 | _ |
| comp29177_c0 | 0.00 | 3.25 | 2.86E-02 | 9.41 | hypothetical protein PRUPE_ppa002277mg [Prunus persica] |
| comp29395_c0 | 0.00 | 20.27 | 1.07E-03 | 10.80 | PREDICTED: uncharacterized protein LOC101261442 [Solanum lycopersicum] |
| comp29487_c0 | 6.47 | 0.00 | 3.33E-02 | -8.09 | _ |
| comp29495_c0 | 0.00 | 9.51 | 4.60E-02 | 9.14 | hypothetical protein PRUPE_ppa009876mg [Prunus persica] |
| comp29885_c0 | 5.63 | 0.00 | 2.76E-02 | -8.33 | hypothetical protein JCGZ_12076 [Jatropha curcas] |
| comp29961_c0 | 0.00 | 16.52 | 1.50E-04 | 10.10 | hypothetical protein EUGRSUZ_F04137 [Eucalyptus grandis] |
| comp30128_c0 | 6.41 | 0.00 | 3.39E-02 | -9.28 | hypothetical protein JCGZ_08793 [Jatropha curcas] |
| comp30431_c0 | 9.18 | 0.00 | 2.67E-02 | -9.50 | LOC100284053 [Zea mays] |
| comp30446_c0 | 6.62 | 0.00 | 5.89E-03 | -9.27 | PREDICTED: 2-methoxy-6-polyprenyl-1,4-benzoquinol methylase, mitochondrial isoform X1 [Cucumis melo] |
| comp30492_c0 | 0.00 | 8.11 | 4.55E-02 | 9.22 | PREDICTED: AT-rich interactive domain-containing protein 2 [Vitis vinifera] |
| comp30524_c0 | 0.00 | 5.07 | 2.17E-02 | 9.59 | PREDICTED: uncharacterized protein LOC100267813 [Vitis vinifera] |
| comp30829_c0 | 0.00 | 5.63 | 2.67E-02 | 8.50 | PREDICTED: vacuolar iron transporter 1-like, partial [Solanum tuberosum] |
| comp30944_c0 | 0.00 | 7.39 | 3.84E-04 | 9.89 | unnamed protein product [Coffea canephora] |
| comp30971_c0 | 0.00 | 9.14 | 2.07E-02 | 10.82 | PREDICTED: protein IQ-DOMAIN 31-like [Vitis vinifera] |
| comp31012_c0 | 0.00 | 8.19 | 2.07E-02 | 9.58 | PREDICTED: SEC12-like protein 1-like [Fragaria vesca subsp. vesca] |
| comp31025_c0 | 0.00 | 5.47 | 2.98E-02 | 9.35 | PREDICTED: cactin-like [Cucumis sativus] |
| comp31096_c0 | 5.32 | 0.00 | 6.72E-03 | -9.06 | PREDICTED: probable beta-1,3-galactosyltransferase 14-like [Solanum lycopersicum] |
| comp32118_c0 | 110.86 | 16.20 | 3.33E-02 | -2.82 | CLP protease proteolytic subunit 2 [Theobroma cacao] |
| comp34915_c0 | 5.54 | 0.00 | 2.81E-03 | -9.46 | PREDICTED: DNA polymerase alpha subunit B [Vitis vinifera] |
| comp36039_c0 | 8.75 | 0.00 | 3.25E-02 | -9.40 | PREDICTED: uncharacterized protein LOC100247481 [Vitis vinifera] |
| comp36781_c0 | 0.00 | 5.13 | 2.86E-02 | 8.37 | PREDICTED: LOW QUALITY PROTEIN: transcription factor bHLH49 [Prunus mume] |
| comp38439_c0 | 6.31 | 0.00 | 7.78E-04 | -9.73 | kinase-like protein TMKL1 precursor [Populus trichocarpa] |
| comp38447_c0 | 11.57 | 0.03 | 6.22E-04 | -8.90 | hypothetical protein JCGZ_23216 [Jatropha curcas] |
| comp39016_c0 | 5.17 | 0.00 | 2.98E-02 | -9.31 | PREDICTED: anthranilate phosphoribosyltransferase, chloroplastic-like isoform X1 [Solanum tuberosum] |
| comp39291_c0 | 0.00 | 11.77 | 1.22E-04 | 10.23 | hypothetical protein JCGZ_14962 [Jatropha curcas] |
| comp39297_c0 | 12.01 | 0.00 | 6.62E-03 | -10.21 | PREDICTED: uncharacterized protein LOC100262433 [Vitis vinifera] |
| comp39345_c0 | 20.28 | 0.00 | 1.55E-02 | -9.83 | unnamed protein product [Coffea canephora] |
| comp39589_c0 | 8.30 | 0.00 | 1.10E-02 | -9.87 | PREDICTED: probable protein arginine N-methyltransferase 1 [Phoenix dactylifera] |
| comp40128_c0 | 0.00 | 4.48 | 1.07E-03 | 9.64 | _ |
| comp40572_c0 | 0.00 | 3.45 | 1.63E-02 | 8.74 | PREDICTED: DNA polymerase alpha subunit B [Vitis vinifera] |
| comp40938_c0 | 0.04 | 47.24 | 6.56E-05 | 11.65 | hypothetical protein CICLE_v10009455mg [Citrus clementina] |
| comp41158_c0 | 0.00 | 5.66 | 6.72E-03 | 9.05 | PREDICTED: probable ATP-dependent RNA helicase YTHDC2-like [Vitis vinifera] |
| comp41423_c0 | 15.66 | 0.00 | 1.05E-02 | -9.99 | PREDICTED: zinc finger A20 and AN1 domain-containing stress-associated protein 8-like isoform 2 [Vitis vinifera] |
| comp41757_c0 | 5.23 | 0.00 | 7.21E-03 | -9.00 | PREDICTED: actin-related protein 2/3 complex subunit 1 [Vitis vinifera] |
| comp42223_c0 | 0.00 | 3.25 | 2.96E-02 | 8.31 | PREDICTED: tRNA-specific adenosine deaminase 1-like isoform X1 [Cicer arietinum] |
| comp42859_c0 | 16.83 | 0.00 | 1.17E-04 | -10.25 | hypothetical protein EUGRSUZ_K00175 [Eucalyptus grandis] |
| comp42996_c0 | 6.65 | 0.00 | 1.89E-02 | -8.65 | hypothetical protein L484_023255 [Morus notabilis] |
| comp43077_c0 | 0.00 | 14.52 | 7.33E-03 | 9.01 | beta carbonic anhydrase 2 [Mesembryanthemum nodiflorum] |
| comp43468_c0 | 1.80 | 48.40 | 1.91E-03 | 4.23 | PREDICTED: ABC transporter C family member 14-like isoform X1 [Citrus sinensis] |
| comp43474_c0 | 0.00 | 6.26 | 4.41E-02 | 9.11 | PREDICTED: aspartic proteinase-like protein 1 [Vitis vinifera] |
| comp43738_c0 | 0.00 | 6.16 | 3.25E-02 | 9.31 | PREDICTED: RAN GTPase-activating protein 2-like [Malus domestica] |
| comp43955_c0 | 0.00 | 8.84 | 3.77E-04 | 10.08 | PREDICTED: UBX domain-containing protein 2-like isoform 2 [Vitis vinifera] |
| comp44109_c0 | 7.28 | 0.00 | 3.83E-02 | -9.27 | PREDICTED: KH domain-containing protein At4g18375 isoform 1 [Vitis vinifera] |
| comp44506_c0 | 0.00 | 1.64 | 3.84E-02 | 7.97 | hypothetical protein EUGRSUZ_I01724 [Eucalyptus grandis] |
| comp44881_c0 | 5.63 | 0.00 | 4.43E-02 | -8.06 | unnamed protein product [Coffea canephora] |
| comp45008_c0 | 2.28 | 0.00 | 3.25E-02 | -9.27 | PREDICTED: uncharacterized protein LOC100255898 [Vitis vinifera] |
| comp45146_c0 | 0.00 | 12.30 | 6.44E-05 | 11.44 | phytochrome B [Vitis riparia] |
| comp45416_c0 | 0.00 | 2.58 | 1.78E-02 | 8.66 | Uncharacterized protein TCM_016013 [Theobroma cacao] |
| comp45804_c0 | 4.98 | 33.21 | 4.31E-02 | 2.79 | PREDICTED: UDP-glucose flavonoid 3-O-glucosyltransferase 7-like [Fragaria vesca subsp. vesca] |
| comp46117_c0 | 7.54 | 0.00 | 1.10E-02 | -8.84 | PREDICTED: uncharacterized protein LOC102590471 [Solanum tuberosum] |
| comp46223_c0 | 6.12 | 0.00 | 9.76E-03 | -9.94 | unnamed protein product [Coffea canephora] |
| comp46592_c0 | 9.78 | 0.00 | 1.17E-04 | -10.19 | hypothetical protein PHAVU_009G159400g [Phaseolus vulgaris] |
| comp46641_c0 | 0.00 | 5.27 | 3.76E-02 | 9.27 | PREDICTED: uncharacterized protein LOC100256014 [Vitis vinifera] |
| comp46943_c0 | 0.00 | 2.73 | 2.63E-02 | 8.50 | an N-terminal calmodulin binding autoinhibitory domain-containing family protein [Populus an N-terminal calmodulin binding au |
| comp47142_c0 | 0.00 | 10.78 | 7.35E-05 | 10.31 | PREDICTED: phosphoenolpyruvate/phosphate translocator 2, chloroplastic-like isoform X1 [Malus domestica] |
| comp47177_c0 | 3.13 | 0.00 | 2.97E-02 | -8.19 | PREDICTED: protein brittle-1, chloroplastic/amyloplastic isoform 1 [Vitis vinifera] |
| comp47347_c0 | 6.73 | 0.00 | 3.91E-02 | -9.22 | PREDICTED: ribosome biogenesis protein WDR12 homolog [Vitis vinifera] |
| comp47427_c0 | 5.75 | 0.00 | 2.11E-02 | -9.08 | hypothetical protein PRUPE_ppa008377mg [Prunus persica] |
| comp48244_c0 | 0.01 | 4.17 | 8.54E-03 | 10.07 | Serine/threonine-protein kinase ATM [Morus notabilis] |
| comp48444_c0 | 6.32 | 0.00 | 3.32E-02 | -9.30 | PREDICTED: SUN domain-containing protein 2-like [Citrus sinensis] |
| comp48592_c0 | 5.05 | 0.22 | 3.25E-02 | -4.85 | transferase, transferring glycosyl groups, putative [Ricinus communis] |
| comp48832_c0 | 0.70 | 21.04 | 5.67E-03 | 6.23 | PREDICTED: uncharacterized protein LOC103498865 isoform X2 [Cucumis melo] |
| comp49054_c0 | 0.00 | 2.72 | 1.89E-02 | 8.61 | PREDICTED: probable NOT transcription complex subunit VIP2-like [Vitis vinifera] |
| comp49332_c0 | 0.00 | 10.73 | 2.85E-02 | 9.48 | hypothetical protein JCGZ_20744 [Jatropha curcas] |
| comp49566_c0 | 0.00 | 3.52 | 3.99E-02 | 9.16 | leucine-rich repeat transmembrane protein kinase [Populus trichocarpa] |
| comp49651_c0 | 0.00 | 2.76 | 3.04E-02 | 8.18 | PREDICTED: 3-hydroxyisobutyryl-CoA hydrolase 1 [Vitis vinifera] |
| comp50456_c0 | 3.72 | 0.00 | 4.86E-02 | -9.02 | PREDICTED: probable ATP-dependent RNA helicase DHX35 [Vitis vinifera] |
| comp50655_c0 | 0.00 | 4.77 | 3.72E-02 | 9.32 | _ |
| comp50965_c0 | 10.24 | 3.59 | 1.17E-02 | -4.59 | PREDICTED: uncharacterized protein LOC100242968 [Vitis vinifera] |
| comp51113_c0 | 10.78 | 0.00 | 3.21E-02 | -9.34 | PREDICTED: adenylyl-sulfate kinase 1, chloroplastic [Vitis vinifera] |
| comp51118_c0 | 5.05 | 0.00 | 9.40E-03 | -10.05 | PREDICTED: probable leucine-rich repeat receptor-like protein kinase At2g33170-like [Vitis vinifera] |
| comp51193_c0 | 27.55 | 0.00 | 3.43E-04 | -11.07 | triose phosphate isomerase cytosolic isoform-like protein [Capsicum annuum] |
| comp51336_c0 | 0.00 | 5.92 | 1.07E-03 | 9.63 | kinase-like protein TMKL1 precursor [Populus trichocarpa] |
| comp51575_c0 | 1.18 | 32.85 | 1.56E-03 | 6.65 | beta-galactosidase 3 [Camellia sinensis] |
| comp51591_c0 | 0.00 | 15.73 | 2.26E-02 | 9.53 | AP-4 complex subunit sigma [Morus notabilis] |
| comp51615_c0 | 5.62 | 0.00 | 4.06E-02 | -9.17 | hypothetical protein POPTR_0004s06800g [Populus trichocarpa] |
| comp52340_c0 | 3.98 | 0.18 | 3.09E-02 | -7.51 | hypothetical protein EUTSA_v10006970mg [Eutrema salsugineum] |
| comp52470_c1 | 0.01 | 16.22 | 4.12E-10 | 9.41 | PREDICTED: uncharacterized protein LOC100807239 [Glycine max] |
| comp52575_c0 | 8.76 | 0.00 | 5.67E-03 | -9.23 | Cyclin p3,2 [Theobroma cacao] |
| comp52647_c0 | 27.54 | 0.51 | 6.50E-07 | -6.87 | PREDICTED: cytochrome P450 83B1 [Vitis vinifera] |
| comp52696_c0 | 0.00 | 3.56 | 3.39E-03 | 10.37 | Regulator of nonsense transcripts 2 isoform 1 [Theobroma cacao] |
| comp53138_c0 | 0.00 | 5.75 | 1.55E-02 | 8.82 | conserved hypothetical protein [Ricinus communis] |
| comp54517_c0 | 0.32 | 6.86 | 1.89E-02 | 5.03 | P-type ATPase of 2 isoform 1 [Theobroma cacao] |
| comp54535_c0 | 0.00 | 5.60 | 3.94E-02 | 9.27 | flavonoid 3'-hydroxylase [Dianthus caryophyllus] |
| comp54706_c0 | 2.46 | 16.37 | 2.24E-04 | 5.54 | hypothetical protein PRUPE_ppa003145mg [Prunus persica] |
| comp54871_c0 | 9.06 | 0.00 | 1.50E-04 | -10.11 | DNA binding protein, putative [Ricinus communis] |
| comp54947_c0 | 4.83 | 0.00 | 1.78E-02 | -8.76 | acyl CoA:diacylglycerol acyltransferase [Tetraena mongolica] |
| comp55674_c0 | 20.29 | 5.44 | 6.72E-03 | -5.28 | hypothetical protein JCGZ_07660 [Jatropha curcas] |
| comp56532_c0 | 11.96 | 0.00 | 4.96E-05 | -11.60 | Purine biosynthesis 4 [Theobroma cacao] |
| comp58949_c0 | 0.00 | 30.53 | 4.72E-06 | 10.66 | Peptidyl-prolyl cis-trans isomerase FKBP12 [Morus notabilis] |
| comp58993_c0 | 0.00 | 6.98 | 6.62E-03 | 9.30 | cytochrome P450, partial [Betula platyphylla] |
| comp59149_c0 | 0.00 | 28.03 | 1.48E-02 | 8.83 | heat shock cognate 70 kDa protein [Phytolacca acinosa] |
| comp59352_c0 | 0.00 | 26.40 | 1.89E-02 | 9.74 | PREDICTED: heavy metal-associated isoprenylated plant protein 26-like [Cucumis PREDICTED: heavy metal-associated isoprenylat |
| comp59368_c0 | 0.00 | 6.37 | 3.67E-02 | 10.24 | RecName: Full=2,3-bisphosphoglycerate-independent phosphoglycerate mutase; Short=BPG-independent PGAM; Short=Phosphoglyceromutase; AltName: Full=PGAM-I [Mesembryanth |
| comp59513_c0 | 43.98 | 0.00 | 2.43E-05 | -11.95 | Hsp90 co-chaperone AHA1, putative [Ricinus communis] |
| comp59826_c0 | 28.62 | 0.00 | 1.15E-08 | -11.21 | PREDICTED: cycloartenol-C-24-methyltransferase isoform 1 [Vitis vinifera] |
| comp60074_c0 | 29.86 | 0.00 | 2.75E-03 | -10.63 | serine-threonine protein kinase, plant-type, putative [Ricinus communis] |
| comp60351_c0 | 0.00 | 18.92 | 1.94E-02 | 9.80 | chalcone isomerase [Garcinia mangostana] |
| comp60504_c0 | 9.17 | 0.00 | 2.64E-04 | -9.97 | O-Glycosyl hydrolases family 17 protein isoform 1 [Theobroma cacao] |
| comp60505_c0 | 2.67 | 0.00 | 3.25E-02 | -9.31 | Leucine-rich repeat containing protein, putative isoform 1 [Theobroma cacao] |
| comp60644_c0 | 0.01 | 28.54 | 3.77E-04 | 11.05 | RecName: Full=50S ribosomal protein L35, chloroplastic; AltName: Full=CL35; Flags: Precursor 5 Chain 5, Homology Model For The Spinach Chloropla |
| comp60790_c0 | 1.07 | 71.68 | 4.86E-05 | 6.02 | polyamine oxidase [Amaranthus hypochondriacus] |
| comp60838_c0 | 20.49 | 0.00 | 4.06E-02 | -9.17 | hypothetical protein JCGZ_17028 [Jatropha curcas] |
| comp60908_c0 | 0.00 | 16.20 | 1.83E-04 | 10.11 | hypothetical protein CISIN_1g030522mg [Citrus sinensis] |
| comp61011_c0 | 0.00 | 21.94 | 2.76E-02 | 8.36 | Bifunctional polymyxin resistance protein ArnA [Morus notabilis] |
| comp61274_c0 | 48.76 | 0.00 | 1.50E-04 | -11.27 | hypothetical protein VITISV_024323 [Vitis vinifera] |
| comp61654_c0 | 0.00 | 5.90 | 2.43E-02 | 8.50 | PREDICTED: uncharacterized protein LOC101206914 [Cucumis sativus] |
| comp61952_c0 | 18.76 | 0.00 | 1.25E-02 | -9.92 | PREDICTED: oxygen-evolving enhancer protein 3-2, chloroplastic [Vitis vinifera] |
| comp62008_c0 | 160.97 | 10.05 | 4.06E-02 | -3.51 | hypothetical protein PHAVU_001G085200g [Phaseolus vulgaris] |
| comp62400_c0 | 16.85 | 0.00 | 3.39E-03 | -10.42 | 12-oxophytodienoate reductase [Hevea brasiliensis] |
| comp62460_c0 | 0.00 | 15.07 | 1.78E-02 | 8.62 | hypothetical protein POPTR_0009s08510g [Populus trichocarpa] |
| comp62782_c0 | 0.00 | 8.10 | 6.57E-04 | 9.88 | Cytochrome P450 [Theobroma Cytochrome P450 [Theobroma cacao] |
| comp63144_c0 | 10.40 | 0.00 | 6.62E-03 | -9.12 | PREDICTED: uncharacterized protein LOC100262661 [Vitis vinifera] |
| comp63290_c0 | 0.00 | 12.81 | 2.75E-03 | 9.40 | PREDICTED: 60S ribosome subunit biogenesis protein NIP7 homolog [Cucumis melo] |
| comp63298_c0 | 0.00 | 21.74 | 5.16E-04 | 9.88 | putative histone deacetylase [Trifolium pratense] |
| comp63429_c0 | 0.00 | 9.21 | 1.05E-02 | 10.06 | Aspartate aminotransferase 3 [Theobroma cacao] |
| comp63571_c0 | 0.00 | 10.12 | 4.06E-02 | 9.21 | PREDICTED: dihydroflavonol-4-reductase [Vitis vinifera] |
| comp63608_c0 | 13.51 | 0.00 | 7.21E-03 | -10.04 | PREDICTED: DNA-directed RNA polymerases I, II, and III subunit RPABC1-like [Vitis vinifera] |
| comp63780_c0 | 0.00 | 26.95 | 3.32E-05 | 10.63 | hypothetical protein POPTR_0006s28280g [Populus trichocarpa] |
| comp63792_c0 | 2.35 | 0.00 | 1.83E-02 | -9.63 | hypothetical protein JCGZ_26756 [Jatropha curcas] |
| comp63895_c0 | 10.35 | 0.00 | 2.98E-02 | -8.25 | PREDICTED: patellin-6 [Vitis vinifera] |
| comp64168_c0 | 0.00 | 5.69 | 3.95E-02 | 8.16 | conserved hypothetical protein [Ricinus communis] |
| comp64249_c0 | 11.20 | 0.00 | 3.21E-02 | -9.34 | MSF1-like family protein [Populus trichocarpa] |
| comp64506_c0 | 4.75 | 0.00 | 3.06E-02 | -8.29 | PREDICTED: p21-activated protein kinase-interacting protein 1-like [Vitis vinifera] |
| comp64781_c0 | 0.00 | 16.17 | 3.09E-08 | 11.10 | hypothetical protein CICLE_v10007800mg [Citrus clementina] |
| comp65301_c0 | 13.61 | 0.00 | 2.97E-02 | -8.23 | Secretory carrier-associated membrane protein [Medicago truncatula] |
| comp65852_c0 | 5.97 | 0.00 | 2.02E-04 | -10.07 | lipoxygenase 2 [Capsicum annuum] |
| comp66404_c0 | 0.00 | 13.01 | 9.40E-03 | 8.93 | ACT domain repeat 8 [Theobroma ACT domain repeat 8 [Theobroma cacao] |
| comp66579_c0 | 0.00 | 10.90 | 1.58E-02 | 9.77 | MtN19-like protein, putative isoform 1 [Theobroma cacao] |
| comp67161_c0 | 0.00 | 9.55 | 1.90E-02 | 8.61 | hypothetical protein L484_022857 [Morus notabilis] |
| comp68942_c0 | 0.00 | 18.40 | 3.25E-02 | 9.27 | unnamed protein product [Vitis vinifera] |
| comp70988_c0 | 10.37 | 0.00 | 4.60E-02 | -9.07 | hypothetical protein CICLE_v10022249mg [Citrus clementina] |
| comp71557_c0 | 0.00 | 13.86 | 2.97E-02 | 8.25 | _ |
| comp72023_c0 | 0.00 | 4.33 | 4.06E-02 | 7.95 | unnamed protein product [Vitis vinifera] |
| comp72268_c0 | 2.62 | 0.00 | 4.55E-02 | -7.82 | PREDICTED: protein FLC EXPRESSOR [Cucumis melo] |
| comp7429_c0 | 0.00 | 9.90 | 1.27E-04 | 10.17 | DNA binding protein, putative [Ricinus communis] |
| comp75372_c0 | 0.00 | 7.40 | 2.97E-02 | 8.20 | unnamed protein product [Vitis vinifera] |
| comp77648_c0 | 13.29 | 0.00 | 4.14E-02 | -7.95 | _ |
| comp77795_c0 | 2.28 | 0.00 | 4.41E-02 | -7.87 | PREDICTED: 3-ketoacyl-CoA synthase 5-like [Glycine max] |
| comp8179_c0 | 8.47 | 0.00 | 2.80E-02 | -8.37 | _ |
| comp8200_c0 | 0.00 | 8.19 | 3.04E-02 | 9.35 | PREDICTED: uncharacterized protein LOC100254717 [Vitis vinifera] |
| comp83980_c0 | 5.94 | 0.00 | 6.03E-03 | -9.12 | PREDICTED: probable ATP-dependent RNA helicase YTHDC2-like [Vitis vinifera] |
| comp84817_c0 | 3.41 | 0.00 | 3.73E-02 | -8.17 | PREDICTED: proline transporter 1-like [Fragaria vesca subsp. vesca] |
| comp8796_c0 | 3.22 | 0.00 | 4.00E-02 | -9.11 | hypothetical protein JCGZ_14506 [Jatropha curcas] |
| comp9379_c0 | 3.92 | 0.00 | 2.97E-02 | -8.27 | hypothetical protein CICLE_v10012636mg [Citrus clementina] |
| comp9658_c0 | 17.98 | 0.00 | 6.72E-03 | -10.19 | PREDICTED: V-type proton ATPase subunit E-like isoform X2 [Phoenix dactylifera] |
| comp9680_c0 | 28.06 | 0.00 | 3.04E-02 | -9.39 | hypothetical protein EUTSA_v10014731mg [Eutrema salsugineum] |

Note: differentially expressed genes (DEGs) were identified by FDR (false discovery rate) <0.05 and |log2FC| ≥1, log2FC: log2 Folder change (Mn/control)

**Table S5.** Information of 351 DEGs in pokeweed under 50 µM Cd treatment.

| Gene_id | Control fpkm | Cd fpkm | FDR | log2FC | NR_Description |
| --- | --- | --- | --- | --- | --- |
| comp101372_c0 | 0.00 | 9.19 | 2.82E-02 | 8.29 | PREDICTED: MATE efflux family protein 9-like [Vitis vinifera] |
| comp10179_c0 | 0.00 | 8.14 | 4.87E-02 | 8.56 | hypothetical protein POPTR_0015s10940g [Populus trichocarpa] |
| comp102869_c0 | 0.00 | 4.17 | 2.38E-02 | 8.01 | hypothetical protein JCGZ_01049 [Jatropha curcas] |
| comp10423_c0 | 0.00 | 24.54 | 1.84E-03 | 9.37 | Chorismate mutase [Morus notabilis] |
| comp10488_c0 | 0.02 | 8.51 | 9.24E-03 | 9.80 | hypothetical protein PRUPE_ppa008539mg [Prunus persica] |
| comp109135_c0 | 4.72 | 0.00 | 3.43E-02 | -7.56 | PREDICTED: NADPH:quinone oxidoreductase [Vitis vinifera] |
| comp109665_c0 | 0.00 | 16.95 | 2.81E-02 | 7.83 | _ |
| comp11132_c0 | 0.00 | 7.23 | 2.59E-02 | 7.78 | PREDICTED: gibberellin-regulated protein 4-like [Vitis vinifera] |
| comp11427_c0 | 8.56 | 0.00 | 1.44E-02 | -8.29 | PREDICTED: uncharacterized protein LOC100266746 [Vitis vinifera] |
| comp11932_c0 | 9.26 | 0.00 | 2.20E-03 | -9.54 | PREDICTED: uncharacterized protein LOC101213822 [Cucumis sativus] |
| comp12319_c0 | 0.00 | 7.72 | 3.96E-02 | 8.84 | YAB2 protein, partial [Solanum virginianum] |
| comp12436_c0 | 0.00 | 3.64 | 4.92E-02 | 8.63 | PREDICTED: synaptotagmin-5-like [Cucumis melo] |
| comp12687_c0 | 0.00 | 3.59 | 2.38E-02 | 7.92 | hypothetical protein EUGRSUZ_A02967 [Eucalyptus grandis] |
| comp12723_c0 | 0.00 | 7.31 | 1.50E-02 | 8.22 | _ |
| comp12730_c0 | 0.00 | 11.60 | 4.56E-03 | 8.93 | _ |
| comp13087_c0 | 0.00 | 3.38 | 1.24E-02 | 8.49 | PREDICTED: uncharacterized protein LOC101300919 [Fragaria vesca subsp. vesca] |
| comp13117_c0 | 0.00 | 4.71 | 4.02E-02 | 7.57 | hypothetical protein POPTR_0796s00200g [Populus trichocarpa] |
| comp13277_c0 | 0.00 | 7.96 | 2.21E-02 | 7.96 | _ |
| comp13322_c0 | 3.26 | 0.00 | 2.82E-02 | -7.77 | hypothetical protein EUGRSUZ_H04934 [Eucalyptus grandis] |
| comp13577_c0 | 0.00 | 6.74 | 8.86E-04 | 9.57 | PREDICTED: RNA demethylase ALKBH5-like [Malus domestica] |
| comp13711_c0 | 0.00 | 5.07 | 3.40E-02 | 7.70 | hypothetical protein EUGRSUZ_J03137 [Eucalyptus grandis] |
| comp13790_c0 | 9.25 | 0.00 | 4.81E-02 | -7.39 | PREDICTED: pantoate--beta-alanine ligase-like [Fragaria vesca subsp. vesca] |
| comp13793_c0 | 0.00 | 6.36 | 2.57E-02 | 8.20 | PREDICTED: TPR repeat-containing thioredoxin TTL1 [Malus domestica] |
| comp13832_c0 | 0.00 | 6.94 | 1.06E-02 | 8.52 | _ |
| comp14033_c0 | 18.64 | 0.00 | 2.48E-02 | -7.95 | _ |
| comp14096_c0 | 2.05 | 0.00 | 3.39E-02 | -7.77 | PREDICTED: benzyl alcohol O-benzoyltransferase-like [Cucumis melo] |
| comp14320_c0 | 0.00 | 8.53 | 4.81E-02 | 8.56 | salt tolerance protein 3 [Beta vulgaris] |
| comp14335_c0 | 0.00 | 3.93 | 1.45E-02 | 8.35 | PREDICTED: calcium-dependent protein kinase 34-like [Cucumis melo] |
| comp14627_c0 | 7.36 | 0.00 | 4.25E-02 | -7.46 | hypothetical protein POPTR_0018s11040g [Populus trichocarpa] |
| comp14751_c0 | 7.59 | 0.06 | 2.82E-02 | -6.27 | SVP4 [Actinidia chinensis] |
| comp14992_c0 | 0.07 | 10.75 | 1.24E-02 | 6.62 | hypothetical protein EUGRSUZ_C02396 [Eucalyptus grandis] |
| comp15304_c0 | 9.24 | 0.00 | 3.02E-02 | -9.04 | hypothetical protein POPTR_0013s05920g [Populus trichocarpa] |
| comp15372_c0 | 0.00 | 3.27 | 2.21E-02 | 8.03 | PREDICTED: UDP-D-xylose:L-fucose alpha-1,3-D-xylosyltransferase MGP4 [Prunus mume] |
| comp15506_c0 | 12.14 | 0.00 | 4.75E-03 | -8.93 | phosphatidylinositol 3- and 4-kinase family protein [Populus trichocarpa] |
| comp15828_c0 | 0.00 | 4.75 | 4.08E-03 | 9.05 | PREDICTED: aspartic proteinase PCS1-like [Malus domestica] |
| comp15993_c0 | 0.00 | 11.23 | 1.53E-03 | 9.55 | PREDICTED: cytochrome P450 86A8-like [Glycine max] |
| comp16043_c0 | 0.00 | 27.97 | 3.78E-03 | 9.24 | _ |
| comp16384_c0 | 0.00 | 2.75 | 3.33E-02 | 7.70 | PREDICTED: uncharacterized protein LOC101496539 [Cicer arietinum] |
| comp16428_c0 | 0.00 | 14.40 | 2.78E-02 | 7.75 | PREDICTED: cysteine-rich receptor-like protein kinase 25-like [Fragaria vesca subsp. vesca] |
| comp17149_c0 | 0.00 | 5.86 | 2.31E-02 | 9.32 | PREDICTED: dihydroflavonol-4-reductase [Vitis vinifera] |
| comp17281_c0 | 13.08 | 0.00 | 3.39E-03 | -9.14 | PREDICTED: uncharacterized protein LOC103710601 [Phoenix dactylifera] |
| comp17344_c0 | 0.00 | 2.85 | 1.44E-02 | 8.35 | isocitrate dehydrogenase, putative [Ricinus communis] |
| comp17454_c0 | 17.70 | 0.00 | 2.10E-02 | -8.12 | _ |
| comp17493_c0 | 0.00 | 22.32 | 6.28E-07 | 10.84 | PREDICTED: uncharacterized protein LOC102628926 isoform X1 [Citrus sinensis] |
| comp17757_c0 | 8.93 | 0.00 | 4.98E-04 | -9.77 | PREDICTED: peptidyl-prolyl cis-trans isomerase CYP38, chloroplastic [Cucumis melo] |
| comp17966_c0 | 20.94 | 0.00 | 3.60E-02 | -10.21 | glucan endo-1,3-beta-D-glucosidase [Beta vulgaris subsp. vulgaris] |
| comp17968_c0 | 10.48 | 0.00 | 4.68E-02 | -9.99 | protoporphyrinogen oxidase (Protox-I) [Spinacia oleracea] |
| comp19069_c0 | 0.00 | 10.30 | 6.74E-05 | 10.31 | PREDICTED: 26S protease regulatory subunit 8 homolog isoform X1 [Cicer arietinum] |
| comp19701_c0 | 24.50 | 0.00 | 4.56E-03 | -10.14 | PREDICTED: 50S ribosomal protein L29, chloroplastic-like [Vitis vinifera] |
| comp19814_c0 | 19.08 | 98.94 | 1.36E-02 | 2.53 | cationic peroxidase [Beta vulgaris] |
| comp20248_c0 | 0.00 | 28.36 | 4.00E-03 | 9.19 | _ |
| comp20500_c0 | 6.68 | 0.00 | 4.99E-02 | -9.74 | Glyoxalase/Bleomycin resistance protein/Dioxygenase superfamily protein isoform 1 [Theobroma Glyoxalase/Bleomycin resistance protei |
| comp20974_c0 | 9.94 | 0.00 | 5.41E-03 | -8.86 | 60S ribosomal protein L31, putative [Ricinus communis] |
| comp21275_c0 | 16.45 | 0.02 | 4.69E-02 | -9.79 | PREDICTED: mitochondrial import inner membrane translocase subunit Tim13 [Vitis vinifera] |
| comp21427_c0 | 0.00 | 8.03 | 7.15E-04 | 10.79 | NADPH cytochrome P450 reductase [Medicago truncatula] |
| comp21592_c0 | 0.00 | 10.91 | 8.29E-05 | 11.36 | Subtilase family protein isoform 1 [Theobroma cacao] |
| comp21811_c0 | 0.00 | 11.44 | 4.25E-02 | 10.05 | Cytochrome C oxidase polypeptide vib, putative [Theobroma cacao] |
| comp21935_c0 | 0.00 | 7.25 | 1.18E-02 | 8.44 | salinity-induced hypothetical protein [Salicornia brachiata] |
| comp23241_c0 | 9.57 | 0.00 | 3.81E-03 | -9.09 | 26S proteasome non-ATPase regulatory subunit 11 [Morus notabilis] |
| comp24058_c0 | 5.76 | 0.00 | 7.53E-03 | -9.90 | bZIP transcription factor family protein 9 [Camellia sinensis] |
| comp24539_c0 | 0.00 | 7.83 | 1.66E-02 | 9.46 | PREDICTED: uncharacterized protein LOC102603859 [Solanum tuberosum] |
| comp24836_c0 | 0.00 | 7.15 | 2.31E-02 | 9.33 | hypothetical protein EUGRSUZ_F01769 [Eucalyptus grandis] |
| comp24902_c0 | 0.00 | 9.55 | 4.25E-02 | 8.74 | hypothetical protein EUGRSUZ_H00678 [Eucalyptus grandis] |
| comp24905_c0 | 2.92 | 0.00 | 4.75E-02 | -8.55 | TFIIB-related protein [Spinacia oleracea] |
| comp24953_c0 | 0.00 | 9.93 | 4.04E-02 | 7.54 | PREDICTED: caffeoylshikimate esterase-like [Solanum tuberosum] |
| comp25002_c0 | 0.00 | 15.15 | 4.00E-03 | 10.24 | hypothetical protein JCGZ_03363 [Jatropha curcas] |
| comp25286_c0 | 21.36 | 0.00 | 5.26E-03 | -10.07 | Uncharacterized protein isoform 1 [Theobroma cacao] |
| comp25373_c0 | 7.28 | 0.00 | 6.01E-03 | -10.05 | cytochrome P450 monooxygenase [Medicago truncatula] |
| comp25571_c0 | 0.00 | 8.20 | 2.81E-02 | 9.16 | hypothetical protein EUTSA_v10000149mg [Eutrema salsugineum] |
| comp25792_c0 | 10.07 | 0.00 | 3.93E-02 | -8.79 | unnamed protein product [Vitis vinifera] |
| comp25974_c0 | 0.00 | 13.39 | 4.00E-03 | 10.25 | PREDICTED: putative clathrin assembly protein At5g35200 [Prunus mume] |
| comp26357_c0 | 0.00 | 11.78 | 1.77E-03 | 10.55 | hypothetical protein JCGZ_22200 [Jatropha curcas] |
| comp26627_c0 | 0.00 | 6.54 | 2.17E-02 | 9.34 | PREDICTED: uncharacterized protein LOC100243295 isoform 1 [Vitis vinifera] |
| comp27039_c0 | 0.00 | 4.77 | 4.69E-03 | 8.97 | High mobility group family isoform 1 [Theobroma cacao] |
| comp27106_c0 | 0.00 | 13.17 | 4.49E-02 | 9.92 | PREDICTED: pheophorbide a oxygenase, chloroplastic-like [Fragaria vesca subsp. vesca] |
| comp27188_c0 | 0.00 | 9.32 | 7.41E-03 | 9.93 | PREDICTED: uncharacterized protein LOC101313313 [Fragaria vesca subsp. vesca] |
| comp27592_c0 | 2.22 | 0.00 | 4.99E-02 | -7.83 | hypothetical protein PRUPE_ppa020891mg [Prunus persica] |
| comp27624_c0 | 0.00 | 12.26 | 4.21E-04 | 10.93 | sugar transporter, putative [Ricinus communis] |
| comp27635_c0 | 0.00 | 10.90 | 1.16E-03 | 9.56 | PREDICTED: uncharacterized protein LOC100259041 [Vitis vinifera] |
| comp27646_c0 | 554.12 | 78.63 | 2.10E-02 | -2.53 | RecName: Full=Lectin-B; AltName: Full=PL-B; Flags: Precursor mitogen PL-B [Phytolacca americana] |
| comp27699_c0 | 3.32 | 0.00 | 3.38E-02 | -8.96 | unnamed protein product [Coffea canephora] |
| comp27729_c0 | 36.74 | 325.52 | 3.62E-03 | 3.19 | _ |
| comp27762_c0 | 0.00 | 10.90 | 4.75E-03 | 10.13 | hypothetical protein JCGZ_01491 [Jatropha curcas] |
| comp27775_c0 | 0.01 | 10.83 | 2.24E-02 | 9.32 | hypothetical protein VITISV_029907 [Vitis vinifera] |
| comp27784_c0 | 14.80 | 117.50 | 1.84E-03 | 3.08 | _ |
| comp27802_c0 | 0.00 | 44.69 | 1.19E-06 | 12.65 | PREDICTED: secologanin synthase-like [Cicer arietinum] |
| comp27941_c0 | 0.00 | 8.88 | 7.31E-04 | 9.67 | PREDICTED: benzyl alcohol O-benzoyltransferase-like [Cucumis melo] |
| comp27955_c0 | 31.12 | 0.00 | 2.69E-03 | -10.40 | Ribosomal protein L25/Gln-tRNA synthetase, anti-codon-binding domain [Theobroma cacao] |
| comp28090_c0 | 0.00 | 4.37 | 4.07E-02 | 8.86 | PREDICTED: F-box/LRR-repeat protein 2-like [Fragaria vesca subsp. vesca] |
| comp28099_c0 | 5.45 | 0.00 | 1.61E-02 | -9.42 | 2-oxoglutarate and Fe(II)-dependent oxygenase superfamily protein isoform 1 [Theobroma cacao] |
| comp28150_c0 | 14.88 | 0.00 | 8.15E-03 | -8.63 | _ |
| comp28168_c0 | 0.00 | 13.11 | 2.59E-03 | 10.40 | hypothetical protein VITISV_027256 [Vitis vinifera] |
| comp28212_c0 | 0.00 | 4.18 | 4.68E-02 | 7.45 | hypothetical protein PRUPE_ppa001771mg [Prunus persica] |
| comp28309_c0 | 7.75 | 0.00 | 5.57E-03 | -9.94 | hypothetical protein PRUPE_ppa007296mg [Prunus persica] |
| comp28315_c0 | 0.00 | 13.27 | 4.43E-05 | 10.26 | mitogen-activated protein kinase 2 [Glycine max] |
| comp28325_c0 | 7.78 | 0.00 | 2.81E-02 | -9.06 | PREDICTED: transcription initiation factor TFIID subunit 7-like [Vitis vinifera] |
| comp28402_c0 | 216.40 | 47.46 | 2.31E-02 | -2.18 | unknown [Medicago truncatula] |
| comp28547_c0 | 0.00 | 24.58 | 1.46E-07 | 10.94 | hypothetical protein PRUPE_ppa009615mg [Prunus persica] |
| comp28573_c0 | 80.46 | 0.34 | 3.17E-11 | -7.61 | hypothetical protein JCGZ_20771 [Jatropha curcas] |
| comp28625_c0 | 15.36 | 0.00 | 2.88E-02 | -9.13 | N-terminal nucleophile aminohydrolases (Ntn hydrolases) superfamily protein [Theobroma N-terminal nucleophile aminohydrolases (Ntn |
| comp28655_c0 | 10.13 | 0.00 | 2.55E-02 | -7.90 | caffeoyl-CoA-O-methyltransferase [Medicago truncatula] |
| comp28708_c0 | 0.00 | 8.89 | 4.87E-04 | 9.74 | _ |
| comp28727_c0 | 0.00 | 19.29 | 2.51E-03 | 10.40 | PREDICTED: glycolipid transfer protein-like isoform X1 [Citrus sinensis] |
| comp28739_c0 | 0.00 | 7.17 | 2.42E-02 | 8.01 | _ |
| comp28757_c0 | 0.00 | 11.60 | 5.41E-03 | 9.10 | hypothetical protein MIMGU_mgv1a014805mg [Erythranthe guttata] |
| comp28784_c0 | 30.27 | 0.00 | 2.67E-06 | -10.70 | _ |
| comp28819_c0 | 0.00 | 4.88 | 2.59E-02 | 8.35 | PREDICTED: uncharacterized protein LOC100242187 isoform 1 [Vitis vinifera] |
| comp28999_c0 | 0.00 | 24.51 | 3.31E-04 | 9.83 | hypothetical protein POPTR_0006s14310g [Populus trichocarpa] |
| comp29056_c0 | 0.00 | 8.05 | 3.71E-04 | 9.84 | NAD(P)-binding Rossmann-fold superfamily protein isoform 1 [Theobroma cacao] |
| comp29255_c0 | 10.93 | 0.00 | 2.94E-02 | -7.74 | _ |
| comp29334_c0 | 0.00 | 11.75 | 5.19E-03 | 10.10 | hypothetical protein JCGZ_20442 [Jatropha curcas] |
| comp29356_c0 | 3.73 | 0.00 | 1.93E-02 | -9.33 | hypothetical protein CICLE_v10025227mg [Citrus clementina] |
| comp29374_c0 | 0.15 | 5.32 | 3.46E-02 | 5.43 | protein binding protein, putative [Ricinus communis] |
| comp29395_c0 | 0.00 | 7.92 | 5.98E-03 | 10.04 | PREDICTED: uncharacterized protein LOC101261442 [Solanum lycopersicum] |
| comp29429_c0 | 0.00 | 9.57 | 4.00E-03 | 9.02 | Polygalacturonase 2 [Theobroma Polygalacturonase 2 [Theobroma cacao] |
| comp29587_c0 | 0.00 | 9.18 | 7.41E-03 | 8.85 | PREDICTED: haloacid dehalogenase-like hydrolase domain-containing protein 3 [Vitis unnamed protein product [Vitis vinifera] |
| comp29690_c0 | 0.00 | 10.39 | 7.15E-04 | 9.68 | hypothetical protein VITISV_014603 [Vitis vinifera] |
| comp29756_c1 | 0.00 | 13.21 | 2.27E-02 | 8.13 | unnamed protein product [Coffea canephora] |
| comp29960_c0 | 225.91 | 38.36 | 6.22E-03 | -2.63 | ribosome inactivating protein [Bougainvillea spectabilis] |
| comp29961_c0 | 0.00 | 11.16 | 2.55E-02 | 9.25 | hypothetical protein EUGRSUZ_F04137 [Eucalyptus grandis] |
| comp30016_c0 | 15.67 | 0.00 | 2.81E-03 | -10.37 | hypothetical protein PRUPE_ppa005898mg [Prunus persica] |
| comp30021_c0 | 25.36 | 0.02 | 3.54E-03 | -10.33 | PREDICTED: uncharacterized protein At4g28440-like [Phoenix dactylifera] |
| comp30446_c0 | 6.62 | 0.00 | 3.39E-03 | -9.30 | PREDICTED: 2-methoxy-6-polyprenyl-1,4-benzoquinol methylase, mitochondrial isoform X1 [Cucumis melo] |
| comp30447_c0 | 7.80 | 0.00 | 5.47E-03 | -8.85 | small basic intrinsic protein 1 [Vitis vinifera] |
| comp30492_c0 | 0.00 | 8.71 | 1.77E-02 | 9.42 | PREDICTED: AT-rich interactive domain-containing protein 2 [Vitis vinifera] |
| comp30524_c0 | 0.00 | 8.13 | 1.22E-02 | 9.62 | PREDICTED: uncharacterized protein LOC100267813 [Vitis vinifera] |
| comp30654_c0 | 20.23 | 0.00 | 2.45E-02 | -10.32 | hypothetical protein MIMGU_mgv1a014862mg [Erythranthe guttata] |
| comp30817_c0 | 0.00 | 8.72 | 3.70E-02 | 7.57 | hypothetical protein JCGZ_26055 [Jatropha curcas] |
| comp30868_c0 | 0.00 | 10.91 | 3.13E-03 | 10.33 | katanin1 [Gossypium arboreum] |
| comp30880_c0 | 0.00 | 1.36 | 3.48E-02 | 7.57 | PREDICTED: glutamate receptor 3.7 [Vitis vinifera] |
| comp30940_c0 | 7.56 | 0.00 | 3.61E-02 | -8.86 | PREDICTED: protein-S-isoprenylcysteine O-methyltransferase A-like isoform X3 [Solanum tuberosum] |
| comp30949_c0 | 42.52 | 0.00 | 8.29E-05 | -11.32 | phosphoenolpyruvate carboxylase, partial [Commicarpus scandens] |
| comp30971_c0 | 0.00 | 15.09 | 4.00E-03 | 11.37 | PREDICTED: protein IQ-DOMAIN 31-like [Vitis vinifera] |
| comp30978_c0 | 29.55 | 0.00 | 1.29E-02 | -9.62 | PREDICTED: MADS-box protein SVP [Vitis vinifera] |
| comp31064_c0 | 0.00 | 33.14 | 2.36E-03 | 9.38 | _ |
| comp31641_c0 | 18.25 | 0.00 | 4.57E-03 | -8.93 | PREDICTED: MADS-box protein SOC1-like [Cicer arietinum] |
| comp32185_c0 | 0.00 | 5.05 | 2.78E-02 | 7.80 | PREDICTED: protein ODORANT1 [Malus domestica] |
| comp33050_c0 | 0.00 | 44.93 | 5.57E-09 | 11.29 | hypothetical protein CICLE_v10028688mg [Citrus clementina] |
| comp33112_c0 | 0.00 | 13.52 | 2.67E-03 | 9.35 | hypothetical protein RCOM_0407930 [Ricinus communis] |
| comp34855_c0 | 6.83 | 0.01 | 4.13E-02 | -9.93 | PREDICTED: thaumatin-like protein 1 [Malus domestica] |
| comp34987_c0 | 0.00 | 7.13 | 4.47E-02 | 8.65 | PREDICTED: vacuolar cation/proton exchanger 3-like [Vitis vinifera] |
| comp35281_c0 | 0.00 | 5.91 | 4.48E-02 | 8.70 | hypothetical protein RCOM_0407930 [Ricinus communis] |
| comp35945_c0 | 3.31 | 0.00 | 2.52E-02 | -8.31 | hypothetical protein VITISV_021046 [Vitis vinifera] |
| comp38591_c0 | 0.00 | 15.17 | 4.29E-03 | 10.18 | PREDICTED: patellin-6-like [Cucumis sativus] |
| comp38866_c0 | 12.97 | 0.00 | 8.86E-04 | -10.70 | hypothetical protein SORBIDRAFT_02g035230 [Sorghum bicolor] |
| comp38995_c1 | 8.58 | 0.00 | 5.98E-03 | -8.81 | FKBP12-interacting protein of 37 kDa, putative [Ricinus communis] |
| comp39020_c0 | 0.06 | 20.21 | 4.81E-02 | 9.85 | PREDICTED: biotin carboxyl carrier protein of acetyl-CoA carboxylase [Vitis vinifera] |
| comp39345_c0 | 20.28 | 0.00 | 7.82E-03 | -9.86 | unnamed protein product [Coffea canephora] |
| comp39528_c0 | 0.00 | 3.84 | 1.78E-02 | 9.50 | hypothetical protein PRUPE_ppa002542mg [Prunus persica] |
| comp39551_c0 | 3.99 | 0.00 | 2.04E-02 | -9.40 | hypothetical protein JCGZ_05724 [Jatropha curcas] |
| comp39633_c0 | 10.72 | 0.00 | 2.00E-02 | -9.49 | Protein MEMO1 [Morus notabilis] |
| comp39805_c0 | 0.00 | 8.93 | 4.22E-02 | 8.78 | Eukaryotic translation initiation factor 3 subunit K isoform 1 [Theobroma cacao] |
| comp39809_c0 | 0.83 | 16.37 | 6.18E-03 | 4.62 | hypothetical protein PRUPE_ppa003333mg [Prunus persica] |
| comp39839_c0 | 0.00 | 5.89 | 1.16E-02 | 9.68 | PREDICTED: serine/threonine-protein kinase-like protein CCR4-like [Solanum lycopersicum] |
| comp40450_c0 | 4.01 | 0.00 | 2.04E-02 | -8.05 | PREDICTED: serine/threonine-protein kinase HT1 [Cucumis melo] |
| comp40938_c0 | 0.04 | 45.15 | 7.42E-05 | 11.30 | hypothetical protein CICLE_v10009455mg [Citrus clementina] |
| comp41158_c0 | 0.00 | 6.04 | 3.26E-03 | 9.16 | PREDICTED: probable ATP-dependent RNA helicase YTHDC2-like [Vitis vinifera] |
| comp41523_c0 | 6.86 | 0.00 | 6.11E-04 | -9.68 | PREDICTED: RNA demethylase ALKBH5-like [Malus domestica] |
| comp41596_c0 | 6.69 | 0.00 | 1.04E-02 | -9.77 | AMP dependent ligase, putative [Ricinus communis] |
| comp41810_c0 | 8.51 | 0.00 | 1.32E-02 | -9.61 | Protein odr-4-like protein [Morus notabilis] |
| comp41879_c0 | 6.85 | 0.00 | 3.52E-02 | -8.99 | hypothetical protein CICLE_v10012200mg [Citrus clementina] |
| comp42084_c0 | 30.37 | 4.35 | 4.49E-02 | -3.30 | hypothetical protein JCGZ_07098 [Jatropha curcas] |
| comp42158_c0 | 1.81 | 0.00 | 2.58E-02 | -7.90 | PREDICTED: uncharacterized protein LOC100248394 [Vitis vinifera] |
| comp42221_c0 | 0.00 | 7.07 | 3.09E-03 | 9.24 | hypothetical protein CARUB_v10004567mg, partial [Capsella rubella] |
| comp42235_c0 | 5.79 | 0.00 | 1.24E-02 | -8.40 | _ |
| comp42386_c0 | 5.87 | 0.00 | 3.38E-02 | -7.77 | _ |
| comp42559_c1 | 0.00 | 1.40 | 4.49E-02 | 7.59 | hypothetical protein JCGZ_16280 [Jatropha curcas] |
| comp42790_c0 | 0.00 | 7.79 | 9.58E-03 | 8.63 | _ |
| comp42825_c0 | 24.80 | 0.00 | 7.06E-08 | -11.16 | ATP-dependent Clp protease proteolytic subunit-related protein 4 [Morus notabilis] |
| comp43095_c0 | 0.00 | 3.00 | 4.79E-02 | 8.66 | Cellulose synthase A catalytic subunit 3 [UDP-forming] |
| comp43474_c0 | 0.00 | 5.08 | 2.89E-02 | 9.13 | PREDICTED: aspartic proteinase-like protein 1 [Vitis vinifera] |
| comp43541_c0 | 0.00 | 3.13 | 2.37E-02 | 7.92 | Alpha/beta-Hydrolases superfamily protein [Theobroma cacao] |
| comp43667_c0 | 5.64 | 0.00 | 3.00E-05 | -10.38 | PREDICTED: uncharacterized protein LOC102605341 isoform X1 [Solanum tuberosum] |
| comp43738_c0 | 0.00 | 11.14 | 5.19E-06 | 10.56 | PREDICTED: RAN GTPase-activating protein 2-like [Malus domestica] |
| comp43820_c0 | 0.49 | 7.06 | 1.49E-02 | 6.79 | hypothetical protein POPTR_0007s03570g [Populus trichocarpa] |
| comp43833_c0 | 4.03 | 0.00 | 3.83E-02 | -8.82 | PREDICTED: ran-binding protein 10 [Prunus mume] |
| comp43955_c0 | 0.00 | 11.38 | 4.56E-03 | 10.16 | PREDICTED: UBX domain-containing protein 2-like isoform 2 [Vitis vinifera] |
| comp43997_c0 | 3.92 | 39.17 | 4.49E-02 | 3.37 | 9-cis-epoxy-carotenoid dioxygenase [Dianthus caryophyllus] |
| comp44045_c0 | 0.00 | 10.93 | 1.38E-02 | 9.71 | hypothetical protein JCGZ_11042 [Jatropha curcas] |
| comp44293_c0 | 20.32 | 0.20 | 1.22E-02 | -7.23 | hypothetical protein EUGRSUZ_J00392, partial [Eucalyptus grandis] |
| comp44377_c0 | 1.53 | 13.04 | 1.22E-02 | 4.97 | ATP synthase subunit O, mitochondrial [Theobroma cacao] |
| comp44419_c0 | 0.00 | 5.16 | 3.42E-02 | 9.00 | conserved hypothetical protein [Ricinus communis] |
| comp44903_c0 | 11.81 | 0.00 | 1.19E-04 | -10.06 | hypothetical protein L484_023285 [Morus notabilis] |
| comp45046_c0 | 0.00 | 4.20 | 3.89E-02 | 8.84 | leucine-rich repeat transmembrane protein kinase [Populus trichocarpa] |
| comp45146_c0 | 0.00 | 7.07 | 3.14E-02 | 10.21 | phytochrome B [Vitis riparia] |
| comp45255_c0 | 0.00 | 2.59 | 7.78E-03 | 8.94 | Uncharacterized protein TCM_021761 [Theobroma cacao] |
| comp45273_c0 | 0.00 | 15.12 | 5.94E-05 | 10.23 | PREDICTED: uncharacterized protein LOC100242207 [Vitis vinifera] |
| comp45650_c0 | 2.40 | 0.00 | 1.47E-02 | -8.31 | PREDICTED: pentatricopeptide repeat-containing protein At3g61520, mitochondrial [Vitis unnamed protein product [Vitis vinifera] |
| comp45704_c0 | 0.00 | 8.99 | 3.17E-03 | 9.26 | hypothetical protein PHAVU_002G046800g [Phaseolus vulgaris] |
| comp45772_c0 | 18.68 | 0.00 | 1.11E-04 | -10.07 | hypothetical protein POPTR_0002s07720g [Populus trichocarpa] |
| comp45873_c0 | 0.00 | 3.24 | 5.41E-03 | 8.88 | PREDICTED: UPF0392 protein RCOM_0530710-like [Fragaria vesca subsp. vesca] |
| comp45902_c0 | 13.30 | 0.00 | 1.39E-04 | -11.19 | unnamed protein product [Vitis vinifera] |
| comp46064_c0 | 8.99 | 0.00 | 5.57E-03 | -8.94 | PREDICTED: casein kinase I isoform delta-like isoform 1 [Vitis vinifera] |
| comp46226_c0 | 1.54 | 10.05 | 5.98E-03 | 3.95 | PREDICTED: structural maintenance of chromosomes protein 1-like [Citrus sinensis] |
| comp46244_c0 | 14.03 | 0.00 | 4.65E-03 | -10.14 | PREDICTED: pre-mRNA-splicing factor syf2 isoform 1 [Vitis vinifera] |
| comp46413_c0 | 0.00 | 4.11 | 2.55E-02 | 7.85 | BAP1 [Vitis vinifera] |
| comp46943_c0 | 0.00 | 2.51 | 1.07E-02 | 8.76 | an N-terminal calmodulin binding autoinhibitory domain-containing family protein [Populus an N-terminal calmodulin binding au |
| comp47142_c0 | 0.00 | 16.62 | 4.66E-06 | 10.69 | PREDICTED: phosphoenolpyruvate/phosphate translocator 2, chloroplastic-like isoform X1 [Malus domestica] |
| comp47347_c0 | 6.73 | 0.00 | 2.48E-02 | -9.25 | PREDICTED: ribosome biogenesis protein WDR12 homolog [Vitis vinifera] |
| comp47566_c0 | 0.00 | 11.18 | 4.00E-03 | 10.21 | PREDICTED: purple acid phosphatase 2 isoform 1 [Vitis vinifera] |
| comp47606_c0 | 0.00 | 5.43 | 4.91E-02 | 8.62 | CBL-interacting protein kinase 7, putative [Theobroma cacao] |
| comp47915_c0 | 0.00 | 4.30 | 1.17E-02 | 9.68 | zinc finger protein, putative [Ricinus communis] |
| comp48017_c0 | 0.00 | 4.23 | 5.69E-03 | 9.12 | Disease resistance protein [Morus notabilis] |
| comp48487_c0 | 1.30 | 0.00 | 4.55E-02 | -7.46 | hypothetical protein JCGZ_20598 [Jatropha curcas] |
| comp48698_c0 | 5.18 | 0.00 | 1.06E-02 | -8.90 | PREDICTED: membrane-associated protein VIPP1, chloroplastic-like [Solanum tuberosum] |
| comp48878_c0 | 7.83 | 0.00 | 3.60E-02 | -8.88 | PREDICTED: mitochondrial import inner membrane translocase subunit TIM23-1-like [Solanum lycopersicum] |
| comp48886_c0 | 0.00 | 4.25 | 3.60E-02 | 7.73 | putative receptor-like protein kinase [Morus notabilis] |
| comp49157_c0 | 7.01 | 0.00 | 8.15E-03 | -9.83 | hypothetical protein PRUPE_ppa001150mg [Prunus persica] |
| comp49348_c0 | 9.82 | 0.00 | 5.39E-04 | -10.77 | unnamed protein product [Coffea canephora] |
| comp49383_c0 | 0.00 | 11.52 | 6.81E-04 | 9.74 | endo-1,4-beta-mannanase [Populus trichocarpa] |
| comp49566_c0 | 0.00 | 3.02 | 2.76E-02 | 9.14 | leucine-rich repeat transmembrane protein kinase [Populus trichocarpa] |
| comp49744_c0 | 0.00 | 6.66 | 4.91E-02 | 9.29 | PREDICTED: UPF0160 protein MYG1, mitochondrial-like [Vitis vinifera] |
| comp50033_c0 | 1.78 | 0.00 | 2.57E-02 | -8.14 | hypothetical protein MIMGU_mgv1a002315mg [Erythranthe guttata] |
| comp50073_c0 | 0.00 | 5.66 | 1.12E-06 | 10.72 | PREDICTED: LOW QUALITY PROTEIN: zinc finger CCCH domain-containing protein 19 [Prunus mume] |
| comp50134_c0 | 5.78 | 0.00 | 3.80E-03 | -10.15 | PREDICTED: proline-rich receptor-like protein kinase PERK10-like [Vitis vinifera] |
| comp50420_c0 | 10.38 | 0.28 | 8.29E-05 | -8.19 | PREDICTED: probable polyol transporter 4 [Vitis vinifera] |
| comp50445_c0 | 3.34 | 0.00 | 1.18E-02 | -8.40 | PREDICTED: aspartic proteinase-like [Solanum tuberosum] |
| comp50655_c0 | 0.00 | 8.32 | 4.34E-05 | 10.29 | _ |
| comp51234_c0 | 5.26 | 0.00 | 3.26E-03 | -10.23 | unnamed protein product [Vitis vinifera] |
| comp51291_c0 | 0.00 | 16.65 | 2.95E-04 | 9.86 | hypothetical protein POPTR_0002s07720g [Populus trichocarpa] |
| comp51575_c0 | 1.18 | 30.04 | 1.10E-11 | 6.75 | beta-galactosidase 3 [Camellia sinensis] |
| comp51575_c1 | 43.49 | 1.62 | 1.54E-05 | -4.89 | beta-galactosidase 3 [Camellia sinensis] |
| comp51591_c0 | 0.00 | 16.09 | 1.19E-02 | 9.67 | AP-4 complex subunit sigma [Morus notabilis] |
| comp51738_c0 | 0.21 | 13.20 | 5.98E-03 | 8.00 | PREDICTED: SWI/SNF complex component SNF12 homolog [Vitis vinifera] |
| comp52170_c0 | 7.91 | 0.00 | 9.84E-03 | -9.76 | hypothetical protein TRIUR3_24923 [Triticum urartu] |
| comp52237_c0 | 44.39 | 0.00 | 3.48E-15 | -12.36 | PREDICTED: protein disulfide-isomerase-like isoform X1 [Citrus sinensis] |
| comp52470_c0 | 22.90 | 0.61 | 3.00E-05 | -5.91 | PREDICTED: uncharacterized protein LOC100807239 [Glycine max] |
| comp52470_c1 | 0.01 | 17.53 | 1.56E-13 | 9.61 | PREDICTED: uncharacterized protein LOC100807239 [Glycine max] |
| comp52538_c0 | 0.00 | 4.09 | 3.73E-02 | 8.85 | unnamed protein product [Vitis vinifera] |
| comp52584_c0 | 32.93 | 0.50 | 4.43E-05 | -5.75 | PREDICTED: MOB kinase activator-like 1-like [Oryza brachyantha] |
| comp52789_c0 | 13.01 | 0.50 | 1.11E-04 | -5.00 | PREDICTED: uncharacterized protein LOC100244469 [Vitis vinifera] |
| comp52792_c0 | 0.00 | 2.32 | 1.93E-02 | 9.47 | PREDICTED: uncharacterized protein LOC102601421 isoform X1 [Solanum tuberosum] |
| comp52842_c0 | 3.81 | 0.37 | 3.38E-02 | -5.75 | PREDICTED: superkiller viralicidic activity 2-like 2-like [Vitis vinifera] |
| comp53045_c0 | 2.11 | 24.92 | 1.65E-02 | 3.49 | _ |
| comp53138_c0 | 0.00 | 5.68 | 4.68E-02 | 8.65 | conserved hypothetical protein [Ricinus communis] |
| comp53370_c0 | 9.48 | 2.18 | 7.53E-03 | -4.47 | PREDICTED: LOW QUALITY PROTEIN: dolichyl-diphosphooligosaccharide--protein glycosyltransferase subunit STT3A [Malus domestica] |
| comp53850_c0 | 6.97 | 0.36 | 7.78E-03 | -6.43 | hypothetical protein JCGZ_08146 [Jatropha curcas] |
| comp53859_c0 | 2.36 | 0.00 | 2.67E-03 | -9.30 | hypothetical protein JCGZ_10050 [Jatropha curcas] |
| comp54535_c0 | 0.00 | 6.24 | 3.39E-02 | 8.99 | flavonoid 3'-hydroxylase [Dianthus caryophyllus] |
| comp54556_c0 | 0.00 | 2.57 | 1.22E-02 | 8.47 | hypothetical protein JCGZ_23897 [Jatropha curcas] |
| comp54759_c0 | 0.49 | 17.88 | 3.56E-04 | 7.29 | PREDICTED: mitochondrial-processing peptidase subunit alpha [Vitis vinifera] |
| comp54996_c0 | 10.39 | 0.50 | 2.81E-02 | -3.96 | Beta-galactosidase 9 [Morus notabilis] |
| comp54996_c1 | 1.23 | 16.84 | 2.03E-03 | 4.41 | hypothetical protein CICLE_v10004268mg [Citrus clementina] |
| comp55393_c0 | 0.26 | 10.66 | 2.12E-03 | 5.20 | prolyl oligopeptidase family protein [Populus trichocarpa] |
| comp55574_c1 | 0.00 | 2.21 | 4.29E-03 | 9.04 | hypothetical protein PRUPE_ppa000916mg [Prunus persica] |
| comp55674_c0 | 20.29 | 3.99 | 3.26E-03 | -5.34 | hypothetical protein JCGZ_07660 [Jatropha curcas] |
| comp56532_c0 | 11.96 | 0.00 | 1.86E-05 | -11.64 | Purine biosynthesis 4 [Theobroma cacao] |
| comp56881_c2 | 3.34 | 35.53 | 3.16E-02 | 3.23 | WRKY transcription factor 1 [Spinacia oleracea] |
| comp56979_c0 | 0.61 | 11.53 | 9.44E-08 | 8.00 | PREDICTED: putative nuclear matrix constituent protein 1-like protein-like [Vitis vinifera] |
| comp57222_c0 | 3.50 | 0.00 | 3.97E-02 | -8.89 | PREDICTED: xyloglucan galactosyltransferase KATAMARI1 [Prunus mume] |
| comp58906_c0 | 0.00 | 11.49 | 3.01E-02 | 9.04 | cyclophilin [Hevea brasiliensis] |
| comp58949_c0 | 0.00 | 35.69 | 4.90E-04 | 10.92 | Peptidyl-prolyl cis-trans isomerase FKBP12 [Morus notabilis] |
| comp58950_c0 | 56.54 | 0.00 | 2.38E-05 | -11.56 | hypothetical protein CICLE_v10029614mg [Citrus clementina] |
| comp58993_c0 | 0.00 | 9.70 | 6.49E-05 | 10.32 | cytochrome P450, partial [Betula platyphylla] |
| comp59032_c0 | 43.85 | 189.33 | 3.42E-02 | 2.22 | PREDICTED: uncharacterized protein LOC101231385 [Cucumis sativus] |
| comp59071_c0 | 61.96 | 263.78 | 4.90E-02 | 2.08 | copper transport protein ATOX1 [Hevea brasiliensis] |
| comp59107_c0 | 0.00 | 28.04 | 1.39E-04 | 11.19 | cystathionine gamma-synthase [Populus tomentosa] |
| comp59149_c0 | 0.00 | 37.01 | 2.92E-03 | 9.25 | heat shock cognate 70 kDa protein [Phytolacca acinosa] |
| comp59170_c0 | 0.00 | 4.98 | 4.81E-02 | 8.62 | PREDICTED: albumin-2-like [Cicer arietinum] |
| comp59284_c0 | 31.85 | 193.34 | 1.32E-02 | 2.44 | hypothetical protein L484_016474 [Morus notabilis] |
| comp59293_c0 | 55.60 | 0.00 | 3.60E-03 | -10.26 | MIP1.3 [Nicotiana benthamiana] |
| comp59352_c0 | 0.00 | 27.80 | 9.72E-03 | 9.72 | PREDICTED: heavy metal-associated isoprenylated plant protein 26-like [Cucumis PREDICTED: heavy metal-associated isoprenylat |
| comp59358_c0 | 23.81 | 206.62 | 5.26E-03 | 2.86 | _ |
| comp59371_c0 | 573.47 | 63.04 | 5.38E-06 | -3.32 | Cu/Zn superoxide dismutase [Salicornia europaea] |
| comp59432_c0 | 15.78 | 0.00 | 1.10E-04 | -10.10 | Cu/Zn superoxide dismutase [Salicornia europaea] |
| comp59433_c0 | 17.36 | 0.00 | 2.14E-03 | -10.46 | hypothetical protein VITISV_037683 [Vitis vinifera] |
| comp59619_c0 | 0.00 | 13.28 | 4.99E-02 | 7.38 | hypothetical protein AMTR_s00077p00042770 [Amborella trichopoda] |
| comp59642_c0 | 450.76 | 83.68 | 2.82E-02 | -2.36 | _ |
| comp59946_c0 | 7.70 | 0.00 | 2.11E-02 | -9.37 | PREDICTED: malate dehydrogenase, glyoxysomal-like [Fragaria vesca subsp. vesca] |
| comp60351_c0 | 0.00 | 22.94 | 3.84E-06 | 10.70 | chalcone isomerase [Garcinia mangostana] |
| comp60523_c0 | 22.75 | 0.00 | 2.00E-02 | -9.71 | Osmotin-like protein [Theobroma Osmotin-like protein [Theobroma cacao] |
| comp60640_c0 | 73.15 | 0.00 | 1.92E-12 | -11.81 | PREDICTED: tubulin alpha chain [Vitis vinifera] |
| comp60644_c0 | 0.01 | 41.05 | 3.00E-05 | 11.55 | RecName: Full=50S ribosomal protein L35, chloroplastic; AltName: Full=CL35; Flags: Precursor 5 Chain 5, Homology Model For The Spinach Chloropla |
| comp60725_c0 | 14.49 | 0.05 | 2.79E-02 | -10.23 | unnamed protein product [Coffea canephora] |
| comp60744_c0 | 32.01 | 0.00 | 4.47E-02 | -8.60 | hypothetical protein POPTR_0009s12150g, partial [Populus trichocarpa] |
| comp60765_c0 | 0.00 | 23.50 | 8.93E-04 | 10.70 | Ribonuclease 1 [Theobroma cacao] |
| comp60790_c0 | 1.07 | 109.25 | 4.82E-06 | 6.78 | polyamine oxidase [Amaranthus hypochondriacus] |
| comp60832_c0 | 0.00 | 32.61 | 6.16E-06 | 10.56 | hypothetical protein POPTR_0016s13650g [Populus trichocarpa] |
| comp60838_c0 | 20.49 | 0.00 | 2.57E-02 | -9.20 | hypothetical protein JCGZ_17028 [Jatropha curcas] |
| comp61225_c0 | 5.80 | 0.00 | 1.24E-02 | -9.72 | Uncharacterized protein TCM_017020 [Theobroma cacao] |
| comp61378_c0 | 18.36 | 0.00 | 1.63E-02 | -8.27 | 14-3-3-like protein B [Morus notabilis] |
| comp61401_c0 | 19.56 | 0.00 | 2.54E-02 | -8.05 | PREDICTED: bifunctional polymyxin resistance protein ArnA-like [Fragaria vesca subsp. vesca] |
| comp61654_c0 | 0.00 | 4.66 | 3.03E-02 | 7.75 | PREDICTED: uncharacterized protein LOC101206914 [Cucumis sativus] |
| comp61768_c0 | 27.59 | 0.00 | 1.62E-04 | -11.05 | cp protein [Celosia cristata] |
| comp61783_c0 | 15.86 | 0.00 | 7.82E-03 | -8.74 | vacuolar H(+)-ATPase subunit B [Suaeda salsa] |
| comp62217_c0 | 0.00 | 22.48 | 3.96E-02 | 10.03 | RNA-binding protein [Mesembryanthemum crystallinum] |
| comp62275_c0 | 8.29 | 0.00 | 4.32E-03 | -9.05 | uncharacterized protein LOC100306063 precursor [Glycine max] |
| comp62308_c0 | 0.02 | 13.57 | 4.13E-02 | 9.99 | pectin methylesterase PME2.1 [Nicotiana tabacum] |
| comp62318_c0 | 0.00 | 24.66 | 2.55E-02 | 9.23 | PREDICTED: probable methyltransferase PMT26 [Phoenix dactylifera] |
| comp62400_c0 | 16.85 | 0.02 | 5.26E-03 | -8.20 | 12-oxophytodienoate reductase [Hevea brasiliensis] |
| comp62453_c0 | 9.84 | 0.00 | 3.14E-02 | -7.65 | PREDICTED: arabinogalactan peptide 14-like [Solanum tuberosum] |
| comp62524_c0 | 23.20 | 0.00 | 2.46E-03 | -10.43 | hypothetical protein CICLE_v10005917mg [Citrus clementina] |
| comp62539_c0 | 12.18 | 0.00 | 1.03E-02 | -9.70 | RecName: Full=Cyanate hydratase; Short=Cyanase; AltName: Full=Cyanate hydrolase; AltName: Full=Cyanate lyase [Medicago truncatula] |
| comp62613_c0 | 36.09 | 0.00 | 5.12E-12 | -11.66 | hypothetical protein CISIN_1g020436mg [Citrus sinensis] |
| comp62616_c0 | 0.00 | 17.14 | 8.53E-04 | 9.60 | PREDICTED: probable carboxylesterase 5-like [Citrus sinensis] |
| comp62914_c0 | 19.99 | 0.00 | 3.56E-04 | -11.01 | putative luminal binding protein 7B4, partial [Tetragonia tetragonioides] |
| comp63078_c0 | 0.00 | 16.97 | 1.28E-02 | 9.62 | hypothetical protein JCGZ_03443 [Jatropha curcas] |
| comp63240_c0 | 11.70 | 0.00 | 2.21E-02 | -9.32 | hypothetical protein POPTR_0005s08690g [Populus trichocarpa] |
| comp63298_c0 | 0.00 | 13.33 | 2.02E-02 | 9.40 | putative histone deacetylase [Trifolium pratense] |
| comp63308_c0 | 13.16 | 0.00 | 2.82E-02 | -9.16 | Protein C9orf74, putative [Ricinus communis] |
| comp63410_c0 | 0.00 | 12.80 | 2.69E-03 | 10.34 | unnamed protein product [Vitis vinifera] |
| comp63608_c0 | 13.51 | 0.00 | 4.32E-03 | -10.08 | PREDICTED: DNA-directed RNA polymerases I, II, and III subunit RPABC1-like [Vitis vinifera] |
| comp63852_c0 | 14.48 | 0.00 | 1.84E-03 | -9.38 | PREDICTED: probable carboxylesterase 5-like [Citrus sinensis] |
| comp64234_c0 | 0.00 | 22.11 | 3.14E-02 | 7.65 | hypothetical protein PRUPE_ppa012538mg [Prunus persica] |
| comp64249_c0 | 11.20 | 0.00 | 2.04E-02 | -9.37 | MSF1-like family protein [Populus trichocarpa] |
| comp64326_c0 | 0.00 | 8.57 | 3.03E-02 | 7.87 | _ |
| comp64461_c0 | 0.00 | 11.25 | 4.64E-02 | 8.65 | Prenylated Rab acceptor protein, putative [Ricinus communis] |
| comp64559_c0 | 9.54 | 0.00 | 3.16E-02 | -9.00 | PREDICTED: TRM112-like protein At1g78190 [Vitis vinifera] |
| comp64566_c0 | 0.00 | 16.83 | 4.04E-02 | 7.68 | putative aconitase, partial [Dimocarpus longan] |
| comp64859_c0 | 2.62 | 0.00 | 2.98E-02 | -9.04 | hypothetical protein CISIN_1g005868mg [Citrus sinensis] |
| comp64973_c0 | 0.00 | 6.24 | 3.46E-02 | 7.57 | hypothetical protein JCGZ_05714 [Jatropha curcas] |
| comp64989_c0 | 8.96 | 0.00 | 3.40E-02 | -8.85 | pyruvate phosphotransferase family protein [Populus trichocarpa] |
| comp65043_c0 | 0.00 | 14.05 | 2.88E-02 | 9.12 | _ |
| comp65323_c0 | 0.00 | 4.06 | 4.11E-02 | 8.82 | PREDICTED: uncharacterized protein LOC103335559 [Prunus mume] |
| comp65478_c0 | 9.32 | 0.00 | 1.85E-02 | -9.44 | hypothetical protein JCGZ_04611 [Jatropha curcas] |
| comp65686_c0 | 8.46 | 0.00 | 7.15E-04 | -9.64 | PREDICTED: uncharacterized protein LOC103328728 [Prunus mume] |
| comp65865_c0 | 10.03 | 0.00 | 3.16E-02 | -7.68 | 14-3-3 protein isoform g [Nicotiana tabacum] |
| comp66360_c0 | 10.40 | 0.00 | 6.22E-03 | -8.78 | hypothetical protein CICLE_v10003706mg, partial [Citrus clementina] |
| comp66404_c0 | 0.00 | 11.72 | 1.10E-02 | 8.66 | ACT domain repeat 8 [Theobroma ACT domain repeat 8 [Theobroma cacao] |
| comp66579_c0 | 0.00 | 19.23 | 1.77E-03 | 10.51 | MtN19-like protein, putative isoform 1 [Theobroma cacao] |
| comp67497_c0 | 9.41 | 0.00 | 4.47E-02 | -9.87 | hypothetical protein PRUPE_ppa015818mg [Prunus persica] |
| comp67570_c0 | 18.07 | 0.00 | 2.22E-02 | -7.95 | _ |
| comp67994_c0 | 12.81 | 0.00 | 1.77E-03 | -9.48 | hypothetical protein POPTR_0001s27370g [Populus trichocarpa] |
| comp69176_c0 | 12.06 | 0.00 | 6.22E-03 | -8.75 | hypothetical protein POPTR_0006s14310g [Populus trichocarpa] |
| comp69745_c0 | 0.00 | 7.09 | 5.26E-03 | 10.10 | hypothetical protein VITISV_028672 [Vitis vinifera] |
| comp70858_c0 | 0.00 | 19.37 | 2.21E-02 | 8.01 | _ |
| comp7231_c0 | 6.46 | 0.00 | 2.91E-02 | -9.06 | hypothetical protein POPTR_0005s26400g [Populus trichocarpa] |
| comp72623_c0 | 10.25 | 0.00 | 1.19E-02 | -9.68 | PREDICTED: uncharacterized protein LOC103415229 [Malus domestica] |
| comp75273_c0 | 0.00 | 8.68 | 2.57E-02 | 7.83 | potassium channel tetramerisation domain-containing family protein [Populus potassium channel tetramerisation domain-containi |
| comp75315_c0 | 13.94 | 0.00 | 4.83E-02 | -8.63 | PREDICTED: proteinase inhibitor type-2 CEVI57-like [Oryza brachyantha] |
| comp75931_c0 | 3.53 | 0.00 | 2.04E-02 | -8.05 | hypothetical protein EUGRSUZ_A02967 [Eucalyptus grandis] |
| comp76542_c0 | 0.00 | 6.99 | 4.51E-02 | 7.59 | _ |
| comp76655_c0 | 0.00 | 15.96 | 2.57E-02 | 7.83 | _ |
| comp76854_c0 | 8.93 | 0.00 | 3.16E-02 | -7.80 | potassium channel tetramerisation domain-containing family protein [Populus potassium channel tetramerisation domain-containi |
| comp77026_c0 | 0.00 | 7.39 | 4.80E-03 | 8.88 | PREDICTED: protein NLP7-like isoform X1 [Glycine max] |
| comp7737_c0 | 16.26 | 0.00 | 4.64E-02 | -8.68 | _ |
| comp7739_c0 | 0.00 | 28.99 | 6.75E-03 | 8.70 | _ |
| comp77815_c0 | 5.06 | 0.00 | 2.57E-02 | -9.20 | CBL-interacting protein kinase [Populus tomentosa] |
| comp78660_c0 | 0.00 | 11.98 | 3.42E-02 | 7.65 | _ |
| comp78909_c0 | 0.00 | 8.36 | 1.26E-02 | 8.39 | Copper transporter, putative [Theobroma cacao] |
| comp8200_c0 | 0.00 | 6.43 | 2.04E-03 | 9.35 | PREDICTED: uncharacterized protein LOC100254717 [Vitis vinifera] |
| comp82893_c0 | 9.43 | 0.00 | 4.99E-02 | -7.39 | _ |
| comp83980_c0 | 5.94 | 0.00 | 3.26E-03 | -9.15 | PREDICTED: probable ATP-dependent RNA helicase YTHDC2-like [Vitis vinifera] |
| comp8796_c0 | 3.22 | 0.00 | 2.57E-02 | -9.14 | hypothetical protein JCGZ_14506 [Jatropha curcas] |
| comp88801_c0 | 0.00 | 2.56 | 4.13E-02 | 7.48 | Serine/threonine-protein kinase HT1 [Morus notabilis] |
| comp9122_c0 | 0.00 | 13.74 | 3.89E-03 | 9.05 | phosphatidylinositol 3- and 4-kinase family protein [Populus trichocarpa] |
| comp9239_c0 | 3.73 | 0.00 | 2.90E-02 | -9.02 | PREDICTED: vesicle-associated membrane protein 727 [Vitis vinifera] |
| comp92889_c0 | 2.48 | 0.00 | 3.13E-02 | -7.77 | hypothetical protein PRUPE_ppa001427mg [Prunus persica] |
| comp96801_c0 | 0.36 | 11.68 | 4.71E-02 | 4.94 | hypothetical protein VITISV_025029 [Vitis vinifera] |
| comp97501_c0 | 0.18 | 14.39 | 2.00E-02 | 6.37 | PREDICTED: MATE efflux family protein 9-like [Vitis vinifera] |
| comp9794_c0 | 5.03 | 0.00 | 4.23E-02 | -7.46 | unnamed protein product [Coffea canephora] |

Note: differentially expressed genes (DEGs) were identified by FDR (false discovery rate) <0.05 and |log2FC| ≥1, log2FC: log2 Folder change (Cd/Control)

**Table S6.** Results of relating epigenetic variation to genetic, H_2_O_2_, MDA, 8-OHdG, the content of Mn and Cd in three cellular components in pokeweed.

|  | MSAP | |
| --- | --- | --- |
|  | r^2^ | P |
| H_2_O_2_ | 0.8010 | *** |
| MDA | 0.5844 | *** |
| 8-OHDG | 0.7645 | *** |
| Cd-F1 | 0.6242 | *** |
| Cd-F2 | 0.8320 | *** |
| Cd-F3 | 0.7156 | *** |
| Mn-F1 | 0.6733 | *** |
| Mn-F2 | 0.6960 | *** |
| Mn-F3 | 0.8669 | *** |

Note: r^2^ represents the determination coefficient, and the *p*-value indicates the significance of the correlation. **: *p*<0.01, ***: *p*<0.001

**Table S7.** Results of relating DEG variation to the eight PCA axes of MSAP profile.

|  | DEG | |
| --- | --- | --- |
|  | r^2^ | P |
| MSAP-PC1 | 0.9235 | ** |
| MSAP-PC2 | 0.7392 | * |
| MSAP-PC3 | 0.0226 | - |
| MSAP-PC4 | 0.0939 | - |
| MSAP-PC5 | 0.0825 | - |
| MSAP-PC6 | 0.0173 | - |
| MSAP-PC7 | 0.1060 | - |
| MSAP-PC8 | 0.0149 | - |

Note: r^2^ represents the determination coefficient, and the *p*-value indicates the significance of the correlation. *: *p*<0.05, **: *p*<0.01
